# A Glance into the Near Future: Cultivated Meat from Mammalian and Insect Cells

**DOI:** 10.1002/smsc.202400122

**Published:** 2024-07-08

**Authors:** Fabiana Giglio, Carmen Scieuzo, Sofia Ouazri, Valentina Pucciarelli, Dolores Ianniciello, Sophia Letcher, Rosanna Salvia, Ambrogio Laginestra, David L. Kaplan, Patrizia Falabella

**Affiliations:** ^1^ Department of Sciences University of Basilicata Via dell’Ateneo Lucano, 10 85100 Potenza Italy; ^2^ Spinoff Xflies S.R.L University of Basilicata Viale dell’Ateneo Lucano 10 85100 Potenza Italy; ^3^ Department of Biomedical Engineering School of Engineering Tufts University Colby Street, 4 Medford MA 02155 USA; ^4^ Department of Relations with the Territory TotalEnergies EP Italia S.P.A Via della Tecnica, 4 85100 Potenza Italy

**Keywords:** cellular agriculture, cultivated meat, insect cell cultures, scaffolds, sustainable foods

## Abstract

The increasing global population and demand for meat have led to the need to find sustainable and viable alternatives to traditional production methods. One potential solution is cultivated meat (CM), which involves producing meat in vitro from animal stem cells to generate products with nutritional and sensory properties similar to conventional livestock‐derived meat. This article examines current approaches to CM production and investigates how using insect cells could enhance the process. Cell sources are a critical issue in CM production, alongside advances in culture media, bioreactors for scalability, and scaffold development. Insect cells, compared to commonly used mammalian cells, may offer advantages in overcoming technological challenges that hinder cell culture development and expansion. The objective of this review is to emphasize how insects, as a cell source for CM production, could offer a more sustainable option. A crucial aspect for achieving this goal is a comprehensive understanding of the physiology of muscle and fat cells. In this work, the characteristics of insect and mammalian cells are compared, focusing particularly on muscle and fat cell development, regulatory pathways, hormonal regulation, and tissue composition. Insect cells are a promising source for CM, offering a sustainable and environmentally friendly alternative.

## Graphical abstract

Growing demand for meat and environmental concerns are driving the search for sustainable alternatives for meat production. Cultured meat from animal stem cells, particularly insect stem cells, is emerging as an efficient, economical, and environmentally friendly option. This option not only meets continuing increase in global meat demand, but also highlights insects as a compelling alternative to conventional practices.

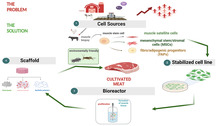

## 1 Introduction

Global meat production is anticipated to increase by almost 44 Mt by 2030, reaching 373 Mt because of rising production as meat prices resume following Covid‐19, according to the publication “OECD‐FAO Agricultural Outlook 2021–2030.”^[^ ^1^ ^]^ Meat consumption is influenced by a variety of factors, including prices, tradition, environmental concerns, animal welfare, and health. Population growth and economic improvement are primary drivers of rising meat consumption, with a projected 14% increase in global meat intake due to an anticipated 11% global rise in population by 2030. Specifically, this increase will be 12% in Latin America, 18% in Asia‐Pacific, 30% in Africa, 0.4% in Europe, and 9% in North America. Economic growth and its structural changes encourage increased meat consumption. According to empirical studies about consumer behavior, higher income drives consumption of more high‐value foods, such as animal proteins, and fewer low‐value products, such as carbohydrates. All these factors have contributed to a dramatic increase in livestock production over the past decade, with growing demand for animal‐based foods among a significant portion of the global population, represented by developing countries.^[^ ^2^ ^]^

This rising demand is problematic since current large‐scale animal farming techniques (generating more than 50% of the world meat supply) are associated with public health risks, environmental degradation, and animal welfare concerns.^[^ ^3^ ^]^ For example, 75% of new infectious diseases in humans are caused by animal sources (zoonotic), primarily as a result of increased human–animal interactions caused by animal husbandry, loss of natural habitats, and the increasing global population.^[^ ^4^, ^5^ ^]^ Animal husbandry, which accounts for 80% of antibiotics used in the United States and 73% of antibiotics sold globally, exacerbates antibiotic resistance, causing increasing risk to human health. Based on the 2020 report by the United Nations Environment Programme on the prevention of future pandemics, the escalation in the worldwide requirement for animal protein products and the unsustainable intensification of agriculture, including the surge of intensive animal agriculture, are two of the seven significant anthropogenic factors that contribute to the emergence of zoonotic diseases. Beyond the dangers to human health, livestock are responsible for 14.5% of all anthropogenic greenhouse gas emissions measured in CO_2_ equivalents. In addition, the production of animal feed has a substantial impact on the environment in terms of land and water use.^[^ ^6^ ^]^ In response to growing concern about the sustainability of large‐scale agriculture, new technologies are emerging for more efficient protein production. One such solution is cultivated meat (CM, also known as cell‐based or cultured meat), which involves the production of meat through in vitro cultivation of animal stem cells, mimicking the natural process of cell growth and division in animals, resulting in a product like traditional meat in terms of nutrition and taste. This is intended to address environmental and animal welfare issues while meeting the needs of a growing global population. Research into CM dates to 2002, when it was observed that the utilization of cultured fish cells could potentially aid in the development of a goldfish muscle explant.^[^ ^7^ ^]^ The first official tasting of CM was in 2013, when Dr. Mark Post's team created a highly publicized hamburger from bovine muscle cells. A growing number of organizations are currently commercializing and scaling CM (at least 70 were reported in mid‐2021).^[^ ^8^ ^]^ For these reasons, in November 2022, the Food and Drug Administration (FDA) assessed the safety of “Cultured chicken (*Gallus gallus*) cell material” provided by Upside Food, a producer of CM. The FDA determined that this was safe and found no evidence that its production process could introduce harmful substances or micro‐organisms into the food.^[^ ^9^ ^]^ Similarly, in March 2023, the FDA evaluated cultured *Gallus gallus* cell material from GOOD Meat,^[^ ^10^ ^]^ a company that already sells CM in Singapore. Singapore was the first country to approve CM production in December 2020, specifically the cultivated chicken bites produced by the US start‐up Eat Just consisting of cultivated chicken cells and plant‐based components.^[^ ^11^, ^12^ ^]^ Food safety regulations vary between countries and regions. In the USA, the FDA oversees food safety, except for meat and poultry, which fall under the jurisdiction of the US Department of Agriculture Food Safety and Inspection Service (USDA–FSIS) under the Federal Meat Inspection Act (FMIA).^[^ ^13^ ^]^ Regarding regulations for cell‐based meat for human consumption, the USDA and FDA issued a joint statement in 2018.^[^ ^14^ ^]^ Under this agreement, the FDA oversees early stages of cell‐based meat development, including cell collection, development, differentiation, and proliferation processes.^[^ ^15^ ^]^ This oversight applies to products derived from cell lines of USDA‐amenable species and requires a USDA mark of inspection.^[^ ^16^ ^]^ Once cells or tissues are ready for harvest, regulatory oversight shifts from the FDA to USDA–FSIS, which ensures the safety, labeling, and overall quality of cell‐based meats. Both agencies inspect production facilities, with USDA–FSIS focusing on final production stages.^[^ ^15^ ^]^ Unlike the United States, CM in Europe falls under either the EU Novel Foods Regulation, which pertains to foods and ingredients not significantly consumed in the EU before May 15, 1997,^[^ ^16^, ^17^ ^]^ or the genetically modified organism (GMO) Legislation (embodied by the GMO Directive^[^ ^18^ ^]^ and GMO Regulation,^[^ ^19^ ^]^ if the initial cell types used are induced pluripotent stem cells (iPSCs).^[^ ^15^, ^20^ ^]^ Member States conduct consultations to determine whether a particular food falls under which regulation, with safety assessments conducted by the European Food Safety Authority.

The main objective of this review is to assess the potential of insect cells as a sustainable and efficient source of CM. This involves a comprehensive comparison of their characteristics with those of mammalian cells, identifying their respective benefits and limitations. In exploring both muscle and adipose tissues, it is essential to recognize the distinct differences between mammals and insects in terms of cellular origins, molecular regulatory pathways, and physiological functions. These comparisons not only illuminate the biological complexity and diversity of these systems in both groups, but also highlight their potential biotechnological applications, including the cultivation of meat.

## 2 Production Process of CM

### 2.1 Structure of Muscle Tissue

**Figure 1:**
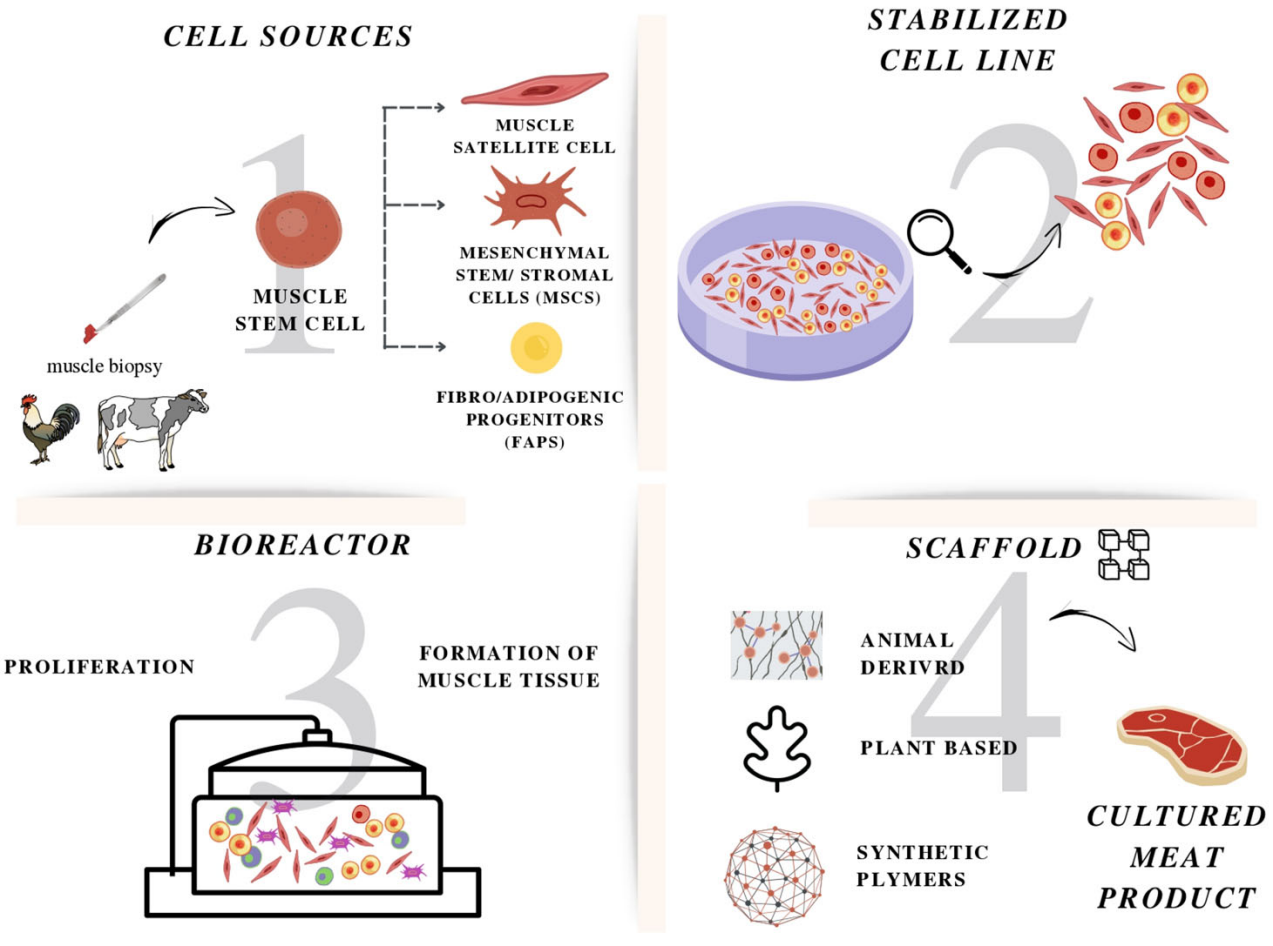
Overview of in vitro CM production. The first step involves harvesting cells from a live animal and obtaining stem cells. The primary cells are then cultivated in an appropriate, nutrient‐rich medium and a cell line is established. Muscle and fat cells are cultured into a bioreactor, a sterile food production facility, to grow and differentiate until muscle tissue is formed. The cells will organize on scaffolds of various origins to form the edible product (Image Created with BioRender.com).

In the production of CM, three fundamental elements play pivotal roles: cells, signals (present in the culture medium), and scaffolds. Cells are the key element, while the culture media provides essential nutrients and small molecules to support cell growth and functions. Scaffolds, made of biocompatible materials, serve as a support to which cells are anchored, facilitating their proliferation and differentiation (**Figure** **1**). The aim of the in vitro CM process is to recreate the tissue structure of animals from different cell sources, primarily focusing on muscle and fat. Myoblastic cells, crucial for muscle tissue formation, can be obtained through various methods. The most common approach involves performing a tissue biopsy of the desired animal or utilizing postmortem tissues. The alternative approach utilizes a source of pluripotent stem cells (PSCs), such as embryonic stem cells (ESCs) or iPSCs. In the first scenario, primary cell cultures can be used directly. In the second, the cells undergo differentiation into mesodermal cells before becoming muscle progenitor cells.^[^ ^21^ ^]^ Myoblast cells fuse naturally in a process known as myogenesis, that is the formation of muscle tissue that occurs particularly during embryonic development.

The architecture of skeletal muscle is a well‐organized distribution of multinucleated contractile muscle cells (also known as muscle fibers) and related connective tissue.^[^ ^22^ ^]^ Muscle development occurs in vivo during embryogenesis with the multiplication of mononucleated myoblasts, which eventually fuse and divide to produce muscle fibers.^[^ ^23^ ^]^ Muscle fibers are functional units surrounded by connective tissue, intramuscular fat, blood vessels, and nerves. The muscle fibers are organized into bundles, and the surrounding connective tissue is composed of endomysium, perimysium, and epimysium. The vessels ensure the transfer of oxygen and nutrients.^[^ ^24^ ^]^ The nutritional value of meat derives mostly from high‐quality protein from muscle that contains all essential amino acids, essential fatty acids, and a variety of vitamins and minerals. Red muscle tissue has more myoglobin and, consequently, more heme iron than white muscle tissue,^[^ ^24^ ^]^ making it a more nutritious source of bioavailable iron. Intramuscular fats contribute to the texture, nutrition, and species‐specific flavor of meat.^[^ ^25^ ^]^ The predominant composition of intramuscular fat consists of adipocytes, which are situated within the interstitial spaces of muscle fibers and fascicles. Intramuscular adipose tissue is composed of various lipid components, including structural lipids, phospholipids, and intracellular lipid droplets located within muscle fibers. In addition, lipids found in fat contain crucial lipophilic vitamins, including A, D, K, and E, alongside essential omega‐3 polyunsaturated fatty acids.^[^ ^26^ ^]^

### 2.2 Cell Sources

**Figure 2:**
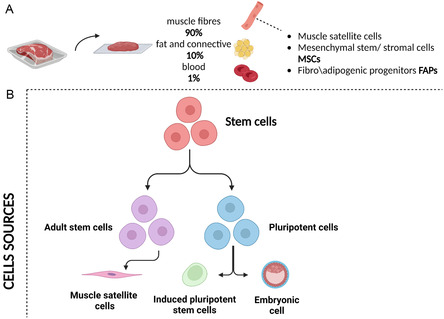
A) The main components of meat. In the microenvironment of muscle fibers, there are progenitor/stem cells such as muscle satellite cells, MSCs, and FAPs. B) Schematic representation of the main sources of stem cells capable of self‐renewal and differentiation into the cells that characterize meat, that is, skeletal myocytes, adipocytes, chondrocytes, and fibroblasts (Image Created with BioRender.com).

The composition of meat typically comprises ≈90% muscle fibers, 10% fat, and connective tissue,^[^ ^24^, ^27^ ^]^ although this varies depending on specific cut of meat and the species it is derived from. Skeletal myocytes are the most numerous cell types in meat, with adipocytes, fibroblasts, chondrocytes, and hematopoietic cells also present and often providing support (**Figure** **2**A,B). Stem cells sourced from a living animal biopsy can be cultivated in vitro to yield substantial cell numbers. These versatile stem cells possess the capability to differentiate into either muscle or fat cells, determined by their specific type.^[^ ^28^ ^]^

Primary cell types for CM production must be capable of adequate self‐renewal and differentiation into the mature cell types that characterize meat. Stem cells are the best option for use as a source of starting cells to satisfy these needs. Adult stem cells and PSCs are the two types of stem cells with the proliferative capacity and differentiation potential necessary for the generation of CM. Traditionally, tissue‐specific stem cells have been the preferred cell source for CM production. They are undifferentiated progenitor cells present in the organs and tissues of animals. Tissue‐specific stem cells are multipotent, meaning they may differentiate into several cell types, the majority of which are relevant to the organ or tissue in which they reside. Within the microenvironment of muscle tissue, the three most frequently encountered types of progenitor/stem cells are muscle satellite cells (MuSCs), mesenchymal stem/stromal cells (MSCs), and fibroadipogenic progenitors (FAPs). The progenitor cells possess the ability to undergo differentiation and give rise to various mature cell types, including but not limited to skeletal myocytes, adipocytes, chondrocytes, and fibroblasts. Muscle satellite cells are a type of stem cell that can be located beneath the basement membrane of muscle fibers. These cells can differentiate into myocytes, which then form multinucleated myotubes that are densely packed into myofibers. MuSCs are one of the most prevalent forms of tissue‐resident adult stem cells,^[^ ^29^ ^]^ and their extraction from animals and maintenance in vitro are well described.^[^ ^30^, ^31^ ^]^ MSCs are commonly found in the bone marrow, but they can also be found in other anatomical locations, such as skeletal muscles, and play a crucial role in muscle regeneration following damage.^[^ ^32^ ^]^ MSCs possess the ability to undergo differentiation into adipocytes, chondrocytes, and fibroblasts.^[^ ^27^, ^30^ ^]^ Mosa Meat, the pioneer of the first cultivated hamburger, has laid the groundwork for cleaner meat alternatives through the use of MSCs.^[^ ^28^, ^33^ ^]^ Various startups, such as Meatable^[^ ^34^ ^]^ and BioTech Foods,^[^ ^35^ ^]^ harness skeletal muscle cells from cattle and/or pigs to create CM products. These products range from minced meat alternatives to delectable and nutritious items like nuggets, hamburgers, and sausages. To achieve the ideal amplification level for processed CM, MuSCs must undergo cell fusion and transition into multinucleated, postmitotic muscle fibers. The differentiation process to myotubes in vitro begins upon MSC exposure to a differentiation medium, typically spanning 3–5 days.^[^ ^36^ ^]^ Controlling MSC activity often involves manipulating extracellular signaling molecules present in the culture medium. Growth factors (GFs) such as insulin‐like GFs (IGF‐1 and ‐2), fibroblast GF (FGF), hepatocyte GF, and cytokines like Tumor Necrosis Factor‐α and leukemia inhibitory factor play pivotal roles in driving MSC activation and proliferation.^[^ ^37^, ^38^, ^39^ ^]^

FAPs, a separate population of MSCs found in the interstitial space of skeletal muscle, are another important cell type for skeletal muscle development and regeneration. FAPs can differentiate into both fibroblasts and adipocytes, which are the connective and fatty tissues found in meat and play a crucial role in myogenic development and organization.^[^ ^31^, ^40^ ^]^ Dedifferentiated fat cells (DFAT) have been identified as a plausible cellular source for the cultivation of adipose tissue. These cells are obtained through the process of dedifferentiation of mature adipocytes. Several commercially accessible immortalized preadipose cell lines exist, such as 3T3‐L1, 3T3‐F442A, and OP9. These exhibit distinct attributes, conducive to the generation of cell culture fat for human consumption. These include a notable capacity for cellular proliferation, resilient differentiation into adipocytes, uniformity in cell populations, uncomplicated maintenance procedures, and comprehensive characterization. Nonetheless, most of these cellular lineages are derived from murine origins, thereby restricting their efficacy in the context of investigating and advancing the production of CM.^[^ ^26^ ^]^

When satellite cells, MSCs, and FAPs are combined, they have the potential to generate all the cell types present in meat. While tissue‐specific stem cells are readily available and capable of differentiating into the necessary mature cell types found in meat, their proliferation and maintenance in vitro are restricted. PSCs have potential as a second cell source for CM production, even though primary tissue‐specific stem cells are a popular cell source. PSCs, such as ESCs and iPSCs, are highly proliferative in culture and can differentiate into every cell type seen in the three primary germ layers (i.e., mesoderm, endoderm, ectoderm). ESCs are sourced from the inner cell mass of the blastocyst, a developmental stage that takes place during the initial phases of mammalian growth. iPSCs are generated by triggering pluripotency genes in somatic cells (Figure 2B).^[^ ^24^, ^41^ ^]^ PSCs derived from non‐muscle sources can be isolated from various domestic animals and harnessed as myogenic cell reservoirs for CM production. Recent advancements include the chemical and genetic modification of pig PSCs to prompt their differentiation into myogenic cells capable of forming embryonic muscle fibers.^[^ ^42^, ^43^ ^]^ Gourmey utilized PSCs to craft cultured foie gras.^[^ ^44^, ^45^ ^]^ However, it is crucial to note that any CM derived from PSCs necessitates clear labeling as genetically modified and must undergo comprehensive safety assessments due to regulatory requirements.^[^ ^43^ ^]^

### 2.3 Cell Immortalization and Differentiation

#### 2.3.1 Rationale for Immortalization of Cultivated Meat Cells

Although primary cell cultures have the benefit of being able to be employed relatively quickly for meat production, they have the disadvantage of being limited in the number of cell divisions they can undergo before senescence or cell cycle arrest. This makes long‐term and commercial production difficult. The use of primary cell cultures necessitates repeated biopsies from live animals, as well as the testing and approval of these biopsies for use in food production.^[^ ^40^ ^]^ Unlike primary cell cultures, immortalized cell lines are not susceptible to senescence and can undergo an endless number of cell divisions. The production of immortalized cell lines is a crucial requirement in the field of cell culture. Even though work on cell lines began more than 50 years ago, there are few cell lines suitable for the cultivation of meat. Indeed, they must conform to particular requirements such as the capacity to proliferate and differentiate (e.g., form mature muscle from muscle cells and accumulate lipids as fat cells) efficiently on an industrial scale, be authorized as safe for ingestion as food, and have the desired properties in terms of flavor, consistency, and nutrition.^[^ ^44^ ^]^ The primary culinary components of animal meat consist of skeletal muscle and adipose tissue. Pertinent cellular entities include satellite cells and stem cells derived from fat tissue, alongside MSCs, versatile fibroblasts, and various types of stem cells.^[^ ^46^, ^47^ ^]^ Myoblast cell lines from model animals are the closest existing cell lines. In addition to consumer impressions, current cell lines lack the flavor, nutrients, and texture associated with meat.[^48^, ^49^] Only recently attempts have been made to establish banks for collecting cell lines appropriate to the development of CM. For example, the Good Food Institute and Kerafast (Boston, Massachusetts) are working together to maintain a bank of terrestrial and aquatic cell lines that can be used for research on CM; however, the number of useful cells remains relatively low.^[^ ^50^ ^]^

#### 2.3.2 Methods to Immortalize Cultivated Meat Cells

Currently, there are three methods to obtain immortalized cell lines: spontaneous immortalization, the development of the telomerase catalytic subunit (TERT), and stimulation via viral genes that inactivate the p53/p14/Rb pathway. Each technique employs telomerase expression, cell cycle inactivation/bypass, or both.^[^ ^40^, ^51^ ^]^ Mammalian cells rarely spontaneously immortalize, and spontaneous immortalization is typically associated with malignancy. This was the first approach used to produce cell lines from the first immortal cell lines recovered from mouse fibroblasts in the 1940s, as well as the *Hela* cell line isolated from cervical cancer cells extracted from Henrietta Lacks. Immortalization can also be triggered by mutagenesis via radiation or chemical carcinogens.^[^ ^52^, ^53^ ^]^ It is also possible to identify clones with immortalization markers, significant TERT expression, or low p15/p16/Rb expression by serial passage of a cell line. The biotechnological company “Future Meat Technologies” has generated a cell line that has spontaneously been immortalized. The present cell line was obtained through the cultivation of fibroblasts that were extracted from a chick embryo, followed by the isolation, concentration, and expansion of colonies of cells that exhibited superior growth characteristics, also known as foci. The colonies underwent expansion to attain a uniform morphology culture, which exhibited the ability to sustain itself beyond 20–30 divisions, exceeding the growth potential of unaltered somatic cells. Each time a non‐immortal cell divides, the telomeres become shorter, up to a point called the Hayflick limit. This makes the telomeres vulnerable to damage and causes senescence. Infection of human fibroblasts and keratinocytes with a retrovirus‐encoding human TERT results in the immortalization of the cell lines. Ectopic expression of TERT in human endothelial cells was also immortalized using plasmid transfection.^[^ ^51^, ^54^ ^]^

Activating the p53/p16/Rb pathways, which bypass the stress response system, is an additional strategy for immortalizing cells. Under normal conditions, p53 is activated in response to DNA damage or other stresses, resulting in cell cycle arrest and apoptosis.^[^ ^55^ ^]^ Rb and p16 activation inhibit the activation of DNA replication by other proteins, resulting in cellular senescence.^[^ ^55^, ^56^ ^]^ Because p16 and Rb are blocked or altered, DNA replication can continue, resulting in cell division.^[^ ^40^ ^]^ TERT expression or p15/p16/Rb inactivation alone is frequently inadequate to immortalize a cell line, indicating that both telomere shortening and the p53/p16 stress response must be avoided. As previously demonstrated, myoblasts must avoid both senescence‐triggering events to attain immortality.^[^ ^57^ ^]^ Upside Foods submitted a patent application in 2016 to immortalize cell lines by overexpressing TERT and utilizing CRISPR‐Cas to suppress the expression of p15 and p16 in chicken skeletal muscle cells.^[^ ^58^ ^]^ TERT overexpression by an ectopic TERT gene enhanced cell proliferative capacity indefinitely, but the deletion of p15 and p16 alone increased cell proliferative capacity. Myogenic cell lines can also be made immortal by expressing genes in a way that skips the shortening of the telomeres and the p16 stress pathway. Other approaches to immortalizing myogenic cell lines can avoid both telomere shortening and stress pathway p16 by ectopically expressing TERT and inhibitors of Rb kinase 4 cyclin‐dependent (CDK4) and cyclin D1.^[^ ^51^, ^59^, ^60^ ^]^

#### 2.3.3 Differentiation in the Context of Cultivated Meat

**Figure 3:**
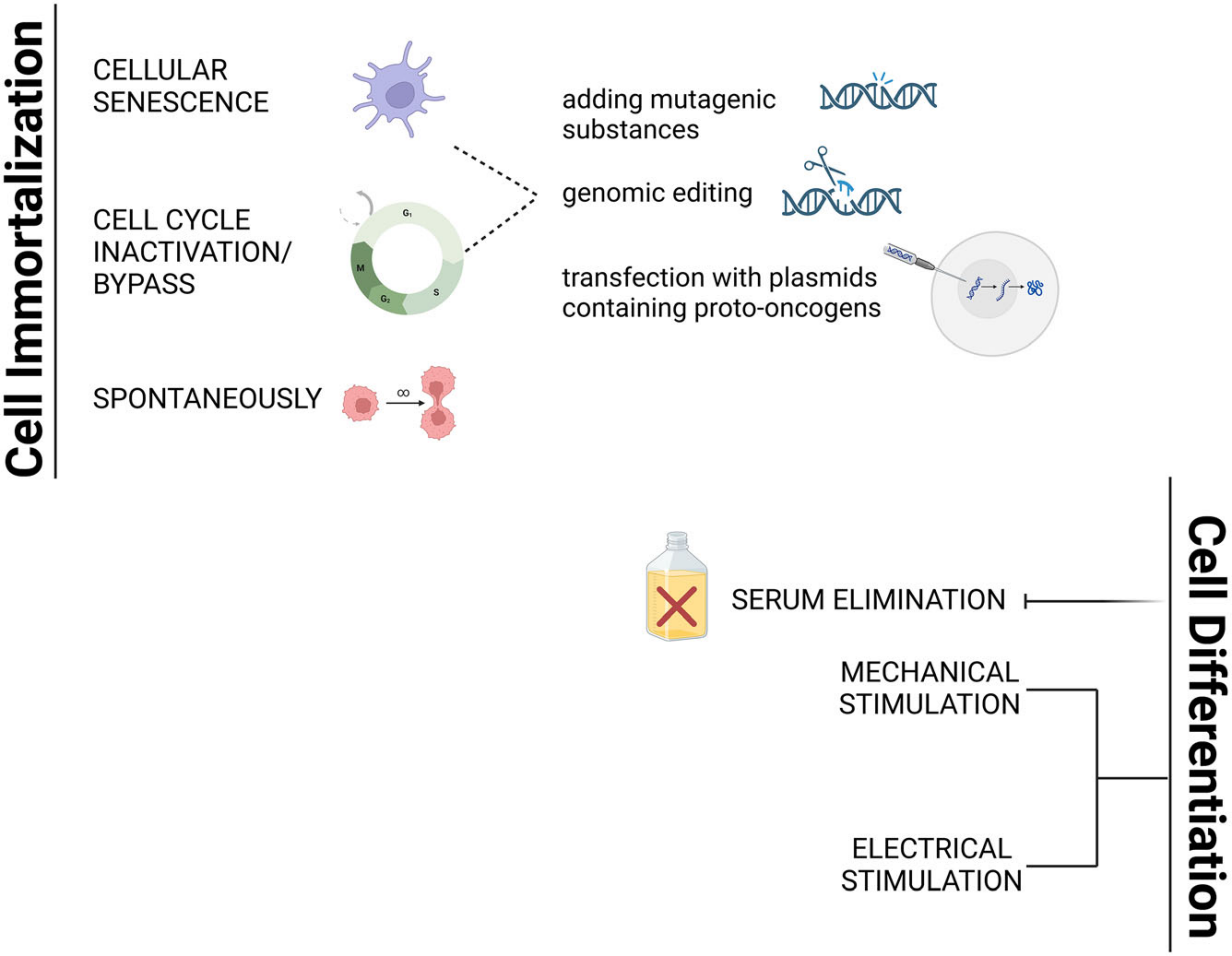
Three methods to obtain immortal cell lines: development of the TERT to avoid cellular senescence, inactivation, or loss of cell cycle checkpoint control by acting on the p53/p14/Rb pathway or spontaneous immortalization (Image Created with BioRender.com).

Muscle cell differentiation occurs in vivo when MuSCs transition from a quiescent to a proliferative state, culminating in myoblast formation.^[^ ^23^ ^]^ From the multiplication of myoblasts, adequate quantities are produced for muscle regeneration, and a portion of these cells also revert to a quiescent state. Because proliferation and differentiation are mutually incompatible processes in in vitro cell growth, cells are typically expanded first and then triggered to differentiate. Differentiation is often achieved by eliminating GFs or introducing differentiation‐promoting proteins. For example, eliminating serum from the culture medium stimulates in vitro differentiation of muscle stem cells, and further maturation can be induced by mechanical and electrical stimulation. Their combined effects especially enhance the early stages of cell proliferation in the absence of a support structure. By acting as a support for propagation and differentiation, scaffolds play a crucial function in terms of mechanical stimulation; their application for cell differentiation mimics the extracellular matrix (ECM)–cell interactions, generally found in vivo via activation of integrin receptors. The effects of electrical stimulation on rat L6 myoblasts were demonstrated using a commercial cell culture stimulation device.^[^ ^61^ ^]^ Electrical stimulation controls myogenic differentiation by reducing the expression of small GTPases^[^ ^62^ ^]^ (**Figure** **3**). When generating CM products, it is likely that muscle cells will be differentiated by the simplest method possible to recapitulate the texture and nutrition of animal‐derived meat.

Adipose tissue is responsible for the regulation and homeostasis of energy metabolism. It is mostly composed of adipocytes surrounded by fibroblasts, fibroblast–preadipocyte cells, endothelial cells, nerve cells, and immune cells.^[^ ^63^ ^]^ For CM production, effective differentiation of adipocytes (i.e., lipid accumulation) is essential. More research is required to create expandable adipogenic stem/progenitor cell lines from meat animal species, food‐grade culture conditions for mature adipocytes, and scalable protocols for creating edible fat tissue, even though the molecular functions and mechanisms of adipocytes have been relatively well studied.

Several approaches have been developed for distinguishing preadipocytes from cells capable of lipid droplet accumulation and exhibiting the morphological and biochemical characteristics of mature white adipocytes.^[^ ^64^ ^]^ Differentiation strategies for preadipose cell lines and primary cultures of fat precursor cells have been created, and the responsiveness of preadipocytes to inducing stimuli can vary greatly. In the presence of fetal bovine serum, preadipocytes spontaneously differentiate into fat cell groups to some extent. The quantity of lipid production may be dose dependently regulated by altering the serum concentration in the growth medium. Inducing substances such as dexamethasone, used to activate the glucocorticoid receptor and 3‐isobutyl‐1‐methylxanthine receptor pathways (IBMX) (or 1‐methyl‐3‐isobutylxanthine, MIX), which is used to stimulate the cAMP‐protein‐dependent kinase, can enhance differentiation.^[^ ^65^ ^]^ In addition, high quantities of insulin have been combined with these inductors. It has been established that insulin/IGF‐1, glucocorticoid, and field‐signaling pathways are involved in the adipocyte differentiation process.^[^ ^66^ ^]^

### 2.4 Culture Media for Cultivated Meat

How to develop and maintain concurrently muscle satellite cells (or, more broadly, muscle precursor cells [MPC]), myoblasts, myocytes (also known as myotubes or myofibers), adipose‐derived stem cells, adipocytes, and fibroblasts is a significant unresolved technical challenge to produce CM.^[^ ^67^ ^]^

Cell proliferation and maintenance depend on a variety of components, such as hormones and GFs. These signals, which are produced in vivo by endocrine glands, bind to specific receptors on the cell membrane or in the cytoplasm and activate the pathways that control cell division and proliferation. Cell culture media contain all the components necessary for cellular survival as well as to stimulate responses such as adhesion, proliferation, and differentiation. In general, basal media consist of carbon and nitrogen sources like glucose, glutamine, and other amino acids; inorganic vitamins and salts; and signaling molecules like GFs.^[^ ^53^, ^67^ ^]^ Formulas for culture media vary according to the application. Recently, several commercially available basal media have become available and usually contain most, but not all, of the ingredients needed for cells to grow, such as glucose, amino acids, and vitamins. Conventionally, basal media formulations are supplemented with complex components of animal origin (i.e., serum) that supply additional nutrients and signaling molecules.^[^ ^53^ ^]^ Eagle minimal essential medium (MEM),^[^ ^68^ ^]^ Dulbecco‐modified Eagle medium (DMEM),^[^ ^69^ ^]^ and Ham F‐12^[^ ^70^ ^]^ are some examples. Serum is the blood fluid collected following blood coagulation; it is rich in proteins, nutrients, GFs, and hormones. Although serum is traditionally used for muscle cell culture,^[^ ^71^ ^]^ it is antithetical to CM production because it is derived from animals. Indeed, a food‐grade medium that is inexpensive, can control large‐scale cell proliferation and differentiation, has acceptable sensory properties, and contains no animal products, is necessary for the effective production of CM.^[^ ^67^ ^]^ Recently, serum‐free media have been created and treated with GFs (particularly insulin, FGF2, and TGF) extracted from animal serum or recombinantly produced.^[^ ^72^, ^73^ ^]^ Essential 8 medium and FGF are two serum‐free media formulations that are commonly used in research to promote stem cell growth. These two formulations have recently shown promise in CM applications, as they support the proliferation of primary bovine myoblasts for at least 6 days.^[^ ^74^, ^75^ ^]^ Many ingredients, including hormones and GFs, are essential for the proliferation and maintenance of cells. In vivo, these signals are generated by endocrine glands and bind to specific receptors on the cell membrane or in the cytoplasm, activating the pathways involved in cell proliferation and differentiation. Insulin and IGFs can promote the development of pluripotent, or adipose‐derived, stem cells into adipocytes when required in a CM application.^[^ ^76^ ^]^ FGF2 is a GF commonly added to muscle cell proliferation media that has a trophic impact on myoblasts by suppressing differentiation.^[^ ^77^ ^]^ Insulin is a commonly used hormone for in vitro maintenance of stem cells. In rat cell cultures, insulin addition stimulated the development of myoblasts.^[^ ^78^ ^]^ The control of catabolism by glucocorticoids influences the proliferation of distinct cell types. In vitro, dexamethasone reduces myoblast doubling time of muscle stem cells, increasing their proliferation potential.^[^ ^79^ ^]^ In addition, dexamethasone treatment promotes satellite cell myogenic differentiation, as evidenced by increased sarcomere formation and enhanced contraction in the resulting myotubes.^[^ ^80^ ^]^ The traditional adipogenic differentiation cocktail includes insulin, dexamethasone, indomethacin, isobutylmethylxanthine, and rosiglitazone.^[^ ^81^ ^]^ Moreover, it has been shown that only two inducers, rosiglitazone and insulin, are needed for serum‐free adipogenesis.^[^ ^82^ ^]^ Tissue development requires many GFs, including TGF‐β, differentiation factors, and bone morphogenetic proteins. In vitro‐cultivated myoblasts treated with TGF‐β are inhibited from developing into myotubes, but their diminished differentiation capacity may be restored once TGF‐β treatment is stopped.^[^ ^83^ ^]^

In vitro‐cultivated animal cells may become contaminated with bacteria, fungi, and yeast. Penicillin, streptomycin, amphotericin B, and gentamicin are some of the antibiotics and antimycotic drugs that have historically been used to treat these contaminations.^[^ ^84^ ^]^ However, antibiotics should generally be avoided during CM production due to their negative side effects on consumers’ sensitivities and they may also contribute to the spread of antibiotic‐resistant bacteria. Both issues could make it more challenging for consumers to accept CM products. Thus, the procedure for producing CM prohibits the use of antibiotic media supplements,^[^ ^67^ ^]^ which are otherwise crucial in the initial phases of primary cell culture development.

For in vitro cell cultivation, it is essential to replicate the animals in an in vivo environment. Incubators are primarily used to maintain a constant temperature, humidity, and pH for cellular homeostasis. The inclusion of sodium bicarbonate buffer in the cell culture medium enables the carbon dioxide (CO_2_) content in the incubator to be regulated, hence preserving the medium pH. The normal incubator settings for culturing stem cells, including muscle cells, are 36.5–37.5 °C, like normal physiological temperature, and 5%–10% CO_2_ for pH regulation.^[^ ^53^ ^]^ Cellular characteristics such as proliferation and differentiation are influenced by variations in cellular respiration and mitochondrial activity due to differences between physiological (1%–6%) and atmospheric (20%) oxygen concentrations. In addition, several recent studies have shown that hypoxia impacts the stemness of muscle stem cells.^[^ ^85^ ^]^ In contrast to atmospheric oxygen concentrations, mouse satellite cells can grow under hypoxic conditions (2% O_2_), proliferating twice as rapidly. When transplanted into cardiotoxin‐damaged muscle, cells grown under hypoxia generated more new muscular fibers than cells cultivated in normoxia.^[^ ^86^ ^]^ Similarly, the myogenic potential of pig satellite cells was improved at low oxygen levels.^[^ ^87^ ^]^

To achieve successful CM production, several key factors must be considered: access to cost‐effective food‐grade media‐component, the capacity to manage cell growth and differentiation on a large scale, desirable sensory characteristics, and the exclusion of animal‐derived components. Valuable insights for developing suitable culture media can be gained from understanding traditional applications of culture media and the metabolic pathways involved in muscle development and protein synthesis. Additionally, strategies employed to enhance media for large‐scale microbial fermentation processes, which yield fundamental chemicals and less valuable food components, can offer valuable guidance.

It is crucial to acknowledge that the culture media used can significantly impact the sensory properties of harvested muscle or meat. Residues from the media present within or on cells could influence flavor, texture, or color. For example, certain amino acids like glutamic acid and asparagine, which contribute to the umami taste in meat, are found in some cell culture media, introducing an additional layer of complexity to media formulation considerations. Recent studies, including preliminary sensory assessments, suggest that laboratory‐scale CM prototypes demonstrate acceptable organoleptic qualities. The process of developing new formulations for CM media begins by identifying key components through experimental or theoretical analysis, followed by optimizing their concentrations. This task is complex due to the large number of components involved, exemplified by DMEM containing between 30 and 52 components, which can lead to intricate interaction effects among them.^[^ ^88^ ^]^ Furthermore, physiological variability of cell lines necessitates reoptimization as processes evolve and new^[^ ^89^ ^]^ components are identified, a common scenario in an industry working with diverse cell lines.^[^ ^90^ ^]^ Therefore, efficient methods for identifying and adjusting concentrations are crucial. Traditionally, media design begins with a one‐factor‐at‐a‐time approach, where each component is assessed individually for its effect on cell response. However, this method overlooks interaction effects, potentially resulting in suboptimal media designs.^[^ ^91^ ^]^ To overcome this limitation, design of experiment (DOE) techniques like factorial, Plackett–Burman, and central composite designs are employed. These methods involve simultaneously changing multiple nutrient concentrations, enabling faster optimization, and have been successfully applied in various industries to characterize and optimize production processes.^[^ ^92^, ^93^, ^94^, ^95^, ^96^ ^]^ DOE experiments are conducted at the extreme ends of the design space to estimate first‐order effects of each media component without interference from others. These experiments are complemented by response surface models (e.g., linear or polynomial models) to predict optimal concentrations and sequentially improve mixtures.^[^ ^97^ ^]^ While effective, these methods can still be experimentally intensive, particularly when optimizing formulations with numerous variables. Alternatively, stochastic optimization methods like genetic algorithms treat media combinations as evolving chromosomes under selection pressures, aiming to maximize fitness (e.g., biomass) of nutrient combinations.^[^ ^98^ ^]^ Modern stochastic methods integrate mathematical surrogate models (e.g., neural networks) to aid in prediction and store information about component effects and interactions, enhancing optimization efficiency over time.^[^ ^99^ ^]^ These advanced optimization techniques have been successful in designing complex microbial media with fewer experiments compared to traditional DOE, demonstrating promising efficiency improvements in media formulation. Continued advancements aim to make these methods more accessible to practitioners, reducing reliance on specialized expertise in artificial intelligence and numerical optimization methodologies.^[^ ^100^ ^]^

### 2.5 Scaffolding

In most cell culture applications for biotechnology, tissue culture flasks and Petri plates are used to establish 2D cell cultures. 2D cell culture is the most common approach for studying cell morphology and the effects of prospective therapies on cell functions. When cells are transplanted from their original tissue into a 2D environment, they typically lose their normal shape, resulting in alterations of the metabolism and gene expression.^[^ ^101^ ^]^ 2D cell culture techniques do not adequately recreate the in vivo environment of the native tissue complex of skeletal muscle, with the absence of cellular connections and communication between cells resulting in slower cell proliferation, less differentiation, and an inability to create epithelial tissue characteristics such as tubular and cystic structures.^[^ ^101^ ^]^ To control the in vitro formation of muscle tissue, scaffolds are often used to simulate the ECM generated by cells and support cell adherence, proliferation, and differentiation. A suitable scaffold for the growth of muscle cells must be edible and cytocompatible and, if utilized in a 3D format must facilitate the exchange of gases, nutrients, and waste to avoid necrosis of the cells. Mimicking the rigidity and protein composition of native ECM helps to replicate the natural microenvironment. This mimicry promotes cell–cell and cell–matrix communication, facilitating cell proliferation and differentiation.^[^ ^102^ ^]^ The scaffolds for CM will likely be modeled in line with tissue engineering (TE) scaffolds, based on biocompatibility, biodegradability, and mechanical properties, while pore size, architecture, and manufacturing methodologies must also be considered.^[^ ^103^ ^]^ Scaffold architecture usually should be porous in order to allow continuous media perfusion, mimicking natural vascularized tissue. Given that muscle tissue (myocytes) constitutes the predominant component of meat, the goal of TE for CM is to produce muscle tissue using cell culture and proper scaffolding.

Scaffold requirements for fat cells are less stringent compared with muscle cell culture, but growth of fat cells within a 3D matrix will likely improve mouthfeel of final products. However, they must still serve as viable substitutes for the typical role played by the ECM. Currently, a variety of scaffolds, including those of synthetic and natural origin, are extensively employed. Polyglycolic acid (PGA) and poly lactic glycolic acid (PLGA) are synthetic materials that are utilized as scaffolds for muscle cells. It is noteworthy that within the category of natural scaffolds, various types exist, such as collagen, collagen‐chitosan hydrogels crosslinked with glutaraldehyde, fibrin, and HYAFF, which is a polymer derived from hyaluronic acid (HA).^[^ ^104^ ^]^

Such scaffolds can be constructed with both synthetic or natural polymers, including those obtained from plants and animals, depending on the origin of the material.^[^ ^105^ ^]^

#### 2.5.1 Natural Polymers

Frequently used scaffolds for skeletal TE are composed of three main groups of natural polymers including proteins (silk, collagen, gelatin, fibrinogen, elastin, keratin, actin, myosin), polysaccharides (cellulose, amylose, dextran, chitin/chitosan, glycosaminoglycans), and polynucleotides (DNA, RNA).^[^ ^106^ ^]^ Natural edible and food safe polymers are commonly used in the production of CM, with plant protein‐based scaffolds being particularly desirable because of the high volume availability, low cost, nutritional value, and cytocompatibility.^[^ ^107^, ^108^ ^]^

#### 2.5.2 Animal‐Derived

Avoiding the use of animal‐derived scaffolds in CM production is an ethical choice, which aims to minimize negative impacts on the lives of animals and promote sustainable and compassionate practices in the food industry. However, it is important to discuss their characteristics by presenting some examples. Collagen is considered an optimal material for scaffolds, that resemble the ECM for human skeletal muscle engineering, and the majority of bioartificial muscles (BAM) are grown on collagen scaffolds.^[^ ^109^, ^110^, ^111^, ^112^ ^]^ Gelatin, a natural component of meat generated when collagen is denatured by processing and heating, has been used to manufacture CM, although it is generally preferred to avoid animal‐derived materials in the process.^[^ ^113^ ^]^ Cultured bovine aorta smooth muscle cells and rabbit skeletal muscle myoblasts replicated several morphological and mechanical properties of natural meat, but lacked the contractile architecture because the gelatin fibers used as the substrate was crosslinked to prevent deterioration.^[^ ^114^ ^]^ Fibrin scaffolds, a naturally occurring fibrous protein that forms blood clots at injury sites, have been used to maximize BAM vascularization. According to the findings, fibrin gel is sufficient for the generation of vascularized BAMs.^[^ ^113^, ^115^, ^116^ ^]^ HA is commonly used in TE because it promotes rapid wound healing and regulates adipogenesis, angiogenesis, and tissue organization in cells. In addition, attempts have been made to replace animal‐sourced HA with endotoxin‐free microorganism‐generated HA via genetic engineering.^[^ ^117^, ^118^ ^]^ Another biopolymer of animal origin used in skeletal muscle scaffolds is chitosan. Chitosan is the main derivative of chitin, a biopolymer found in the exoskeleton of crustaceans and insects and in the cell walls of fungi.^[^ ^119^ ^]^ Currently, the main source of chitin is from crustaceans, but because of limitations linked to seasonality and the poor sustainability of crustacean farming, alternatives are needed. Insects, particularly bioconverters, represent a new alternative and more sustainable source of chitin and chitosan. Indeed, bioconverter insect farms, aimed at the production of animal feed and organic byproduct management (using as insect feed), have spread worldwide, generating huge amounts of insect chitin, mainly derived from pupal exuviae and dead adults. The first characterization of insect chitin and chitosan showed a high degree of similarity with crustacean counterparts, providing a good starting point to use insect biopolymers in the same applications already tested using crustacean sources, including scaffolds.^[^ ^120^, ^121^ ^]^

#### 2.5.3 Plant‐Derived

Plant‐derived scaffolds (e.g., zein, soy protein, wheat gluten) are of interest for CM researchers because of their biodegradability and edibility. Occasionally, these scaffolds may also impart nutritional value and texture to CM.^[^ ^122^ ^]^ One limitation of plant‐derived scaffolds is their lack of mechanical properties; however, this can be remedied by crosslinking. Physical crosslinking, such as UV or thermal processes, are commonly employed. Otherwise, ingestible or FDA‐approved enzymatic or chemical crosslinkers like citric acid, sodium hydroxide, sodium phosphates, or transglutaminase can be utilized to alter the properties of plant‐derived scaffolds, enhancing their mechanical strength to support cell growth. The choice of crosslinkers depends on many factors, such as the base polymeric material, the scaffold architecture, the synthetic process, and cell culture conditions.^[^ ^123^, ^124^ ^]^

Proteins derived from plants can be converted into fibers, films, and hydrogels. In addition, they are readily accessible and reasonably priced. Soy and zein‐derived proteins are commonly used in the production of scaffolds. Soy protein is beneficial for TE since it is biocompatible and shares biochemical properties with the ECM.^[^ ^125^ ^]^ Textured soy protein is favorable for cell adhesion, proliferation, and differentiation of bovine cells and has been used as an edible scaffold to generate cow muscle tissue.^[^ ^126^ ^]^ Soy protein has also been combined with other natural polymers such as chitosan and cellulose and demonstrated favorable adherence and proliferation of multiple cell types (L929, Schwann cells, and human MSCs).^[^ ^127^, ^128^, ^129^ ^]^ Corn zein protein is being investigated for medical applications due to its adaptability and biocompatibility.^[^ ^130^, ^131^ ^]^ It is soluble in ethanol, which facilitates electrospinning and the formation of nanofibers, and nontoxic crosslinking enables fibroblast cell adhesion and growth on electrospun scaffolds.^[^ ^132^ ^]^ Zein scaffolds have also been shown to increase the adhesion, proliferation, and differentiation of human MSCs.^[^ ^133^ ^]^

#### 2.5.4 Polysaccharides

In TE applications, biocompatible polysaccharides derived from plants, such as cellulose and starch, have been used. Cellulose, a linear polysaccharide, is considered as the most sustainable material due to the inexhaustible supply from plant cell walls. Cell culture research has employed a range of cellulose fibers.^[^ ^134^, ^135^ ^]^ However, cellulose is non‐degradable in the human body. Pectin, a natural polysaccharide derived from plant cell walls, provides useful properties as an artificial ECM.^[^ ^136^ ^]^ Pectin/carboxymethyl cellulose/microfibrillated cellulose (pectin/CMC/MFC) scaffolds with different concentrations of MFC (0–0.4%) support NIHT3 fibroblast cell survival.^[^ ^137^ ^]^ Starch is typically blended with synthetic polymers to increase its mechanical and structural qualities as a scaffolding material.^[^ ^136^ ^]^ Alginate, derived from brown seaweeds like *Laminaria hyperborea* and Lessonia, is widely present in coastal waters globally and holds promise as a scaffold material. It is a biocompatible, nontoxic, nonimmunogenic, and biodegradable biopolymer that is economically viable and easily manufacturable. Alginate can be converted into a hydrogel by crosslinking with bivalent cations such as calcium ions (Ca^2**+**^), making it suitable for applications in various fields including food due to its safety profile. However, its negative charge impedes natural cell adhesion, limiting its use in specific applications. To address this, RGD‐modified alginate gels are commonly used as in vitro cell culture platforms, allowing control over myoblast phenotypes. Myoblast adhesion and proliferation on RGD‐modified alginate gels surpass those on unmodified gels. Moreover, the delivery of VEGF and IGF‐1 from alginate gels regulates angiogenesis and myogenesis, facilitating muscle regeneration. Despite its suitability for cell–CM scaffolds, alginate poor cell adhesion remains a challenge, limiting its use to specific applications. Addressing this, researchers achieved 82% cell adhesion coverage by controlling the structure during alginate ionic crosslinking. After an 11‐day culture period, they evaluated cell adhesion, differentiation, and network formation, observing a 12.7% increase in cell growth. Finally, a hybrid cell–CM product was created by blending mycelium‐derived single‐cell protein with cell–CM, yielding an edible, cost‐effective product with desirable texture.^[^ ^138^ ^]^

Agarose, derived from marine red algae, is a natural polysaccharide highly valued in biomedical applications, due to its unique ability to form thermoreversible gels. However, unmodified agarose lacks the optimal cell adhesion properties required for effective TE and cell culture applications.^[^ ^139^ ^]^ To overcome this limitation, researchers have implemented chemical modifications, such as carboxylation via TEMPO‐mediated oxidation under alkaline conditions.^[^ ^140^ ^]^ This approach introduces carboxyl groups onto the agarose backbone, significantly enhancing cell adhesion and bioactivity. The introduction of carboxyl groups transforms the surface properties of agarose, promoting cell adhesion, proliferation, and differentiation through improved protein absorption. Additionally, the conjugation of dopamine to carboxylated agarose further enhances cell adhesion, leveraging dopamine adhesive characteristics inspired by marine mussel proteins.

The chemical modifications of agarose were meticulously characterized using advanced analytical techniques including FT‐IR, 13C NMR, and gel permeation chromatography, confirming the successful integration of carboxyl and dopamine functionalities. In vitro cell culture experiments have demonstrated that these modifications substantially enhance the bioactivity of agarose, making it a promising scaffold material specifically tailored for CM production.^[^ ^141^ ^]^

In summary, the strategic modification of agarose with carboxyl and dopamine functionalities represents a significant advancement in scaffold design, particularly crucial for CM production. This innovative approach supports the development of TE scaffolds that facilitate robust cellular adhesion, growth, and differentiation, thus advancing the field of alternative protein production.

#### 2.5.5 Decellularized Plant Scaffolds

As an alternative to synthetic polymers or animal‐derived scaffolds, the cellulose skeleton of plant tissue can be employed as an affordable scaffold for mammalian cells following decellularization.^[^ ^142^ ^]^ Cellular content is removed from the natural plant material to create an acellular, 3D scaffold that preserves its structural, chemical, and mechanical cues via chemical, physical, or enzymatic methods (trypsin, nucleases, hypo/hypertonic solutions, detergents, solvents). After that, this scaffold may be repopulated with animal cells to create tissue‐engineered constructions for a variety of uses.^[^ ^143^ ^]^ Natural topographies in decellularized plant tissue scaffolds are capable of simulating some of the in vivo features of matrices. However, decellularized plant scaffolds lack a variety of metabolic signals, found in the natural environment, that are necessary to mammalian development. In order to customize these scaffolds for certain cell types, biofunctionalization or coating with functional surface proteins may be required.^[^ ^144^ ^]^ Decellularized plant scaffolds, such as those comprising jackfruit, spinach leaves, and broccoli, have been examined as prospective scaffolds for the regeneration of vascularized tissue mass, utilizing the existing structure to provide perfusion during cell culture.^[^ ^145^, ^146^ ^]^ If the decellularization procedure is nontoxic, it could be used to manufacture CM with structure and lend texture to the final product.^[^ ^122^ ^]^

#### 2.5.6 Synthetic Polymers

To develop a scaffold for CM, the components must either be edible or biodegradable without creating toxic byproducts.^[^ ^122^ ^]^ Otherwise, the cells must be separated from the scaffold. Synthetic polymers most often used in TE are copolymers of polylactic acid (PLA), polyglycolic acid (PGA), and polylactic glycolic acid (PLGA). These are polymers that can be absorbed by living organisms or break down hydrolytically.^[^ ^147^, ^148^ ^]^ PLA has emerged as a crucial material in TE due to its ability to replicate the physical characteristics of the human ECM. PLA nanofiber nonwovens, particularly those created through electrospinning, have garnered significant interest for their potential as TE scaffolds. However, recent studies have shifted focus toward melt‐blown PLA fabrics as alternative scaffolds. These fabrics can be tailored with varying crystallinities, tensile moduli, and pore diameters to mimic specific tissue properties. In a recent study, melt‐blown PLA nonwovens were engineered to resemble human dermis, showing promising outcomes when tested with human dermal fibroblasts over various time frames. Results demonstrated robust cellular attachment, proliferation, and migration, along with cellular penetration through the scaffold thickness. These findings suggest that melt‐blown nanofiber nonwovens hold substantial promise as TE scaffolds, potentially opening new avenues for research in this dynamic field. In another advancement, 3D printing technology has been employed to create patient‐specific scaffolds using PLA‐based materials like PLA‐Baghdadite (Bgh). Following fabrication, these scaffolds were treated and coated with chitosan (Cs)‐vascular endothelial GF (VEGF) or lyophilized Cs‐VEGF to enhance their properties. The coated scaffolds exhibited superior porosity, compressive strength, and elastic modulus compared to traditional PLA samples. Importantly, these scaffolds were found to promote osteogenic differentiation when cultured with rat bone marrow‐derived MSCs, showcasing their potential for bone healing applications. Moreover, innovative scaffold design strategies have been explored using PLA, such as directional porous structures fabricated via ice templating and phase inversion techniques. These scaffolds were engineered to accelerate bone repair by facilitating the growth and proliferation of bone cells. The study showcased the scaffold biocompatibility, mechanical properties, and efficacy in promoting bone regeneration in animal models with large‐sized defects. Previously, edible films formed of PLA have been obtained from dairy waste (via use in fermentation to generate lactic acid that is subsequently polymerized).^[^ ^149^, ^150^ ^]^

However, these materials should have a minimal environmental effect in line with the objectives of CM, and, above all, it could not be a good model for CM scaffold due to its animal origin.^[^ ^151^, ^152^ ^]^ Synthetic polymers often lack biological activity when compared to natural polymers. Hybrid natural–synthetic scaffolds may be useful to satisfy the criteria for CM scaffolding.^[^ ^153^ ^]^

If the scaffold is included in the final food product, the fabrication process and outcomes must be safe for ingestion. The texture, digestion, cooking, water‐binding capacity, and flavor of scaffolds for CM must be considered, particularly in ways that are different than for medical scaffold designs. Ensuring suitability for human consumption as a food ingredient involves a comprehensive approach. This includes not only nutritional analysis, but also mechanical testing to assess texture (such as Warner–Bratzler shear force, water‐holding capacity, and cooking loss). The morphology of a 3D scaffold may be optimized, including fiber size, surface topology, porosity, and pore alignment.^[^ ^154^ ^]^

### 2.6 Bioreactors

Bioreactors are critical for cell expansion and provide stimulation and capacity to scale up cell sources to produce CM. A bioreactor is a container that provides a controlled environment for the growth and development of its cellular contents. A bioreactor maintains the proper biological conditions for cells and culture media, including aiding nutrient transport and cell expansion and differentiation by stirring or stimulating the cells. The classification of bioreactor types is based on the method of medium input into the bioreactor main vessel: batch, fed batch, and continuous.^[^ ^155^ ^]^ A batch bioreactor is a chamber that contains a predetermined volume of growth medium and operates by cultivating cells until they reach their maximum density, at which point they are harvested for utilization or transferred to a larger bioreactor.^[^ ^156^ ^]^ A fed‐batch bioreactor, also known as a semicontinuous bioreactor, has an inlet channel for providing fresh media to the cells at predetermined time intervals chosen to maximize growth. In the absence of a connector to remove conditioned media and cellular products that collect during culture, a fed‐batch bioreactor can also increase volume over time.^[^ ^157^ ^]^ This distinguishes fed‐batch bioreactors from the last major category, continuous. In the production of CM, the preference is often given to fed batch or continuous medium introduction. This approach supports the handling of substantial media volumes, is amenable to automation, and facilitates the recycling of conditioned medium.^[^ ^158^ ^]^ In addition to classification based on medium intake and removal, bioreactors may also be classified based on mixing of internal contents. The bioreactor mixes contents to promote growth and development of the cells. Mechanical bioreactors achieve mixing by agitators or impellers, and these are the most frequently used bioreactors for bioprocess development. The most common mechanical bioreactors are stirred tank bioreactors, which employ an impeller to induce convective flow and facilitate nutrient circulation and diffusion inside the vessel. For bioprocess scaling, stirred tank bioreactors have been the most widely used and since they are well established and scalable, they may be the most suitable bioreactor type for scaling the production of CM.^[^ ^159^ ^]^ Spinner flasks may create turbulent flow that is not favorable to cell multiplication, and the direct contact of cells with the propeller may cause damage. For mammalian cell growth, a continuous stirred tank reactor, which combines continuous medium input with a stirred tank bioreactor system, has been extensively used. Another example of mechanical bioreactor is the rotating‐wall vessel bioreactor that rotates the bioreactor primary vessel around its central axis to dynamically cultivate the vessel contents in suspension.^[^ ^160^ ^]^ Rotating‐wall vessel systems have the advantage of minimum shear stress and may facilitate the formation of 3D aggregates. Nonetheless, several cell types have elevated apoptotic rates early in culture using these systems. Rotating‐wall vessel systems employ batch culture, but perfusion can be incorporated to automate the operation.^[^ ^161^ ^]^ A mechanically active bioreactor system is the last prevalent mechanical bioreactor type. The bioreactor employs a regulated application of mechanical force to cells or tissue scaffolds, specifically using dynamic compression. This stimulation promotes cellular growth by simulating the natural developmental environment and can strengthen and align cells or scaffold structures.^[^ ^162^ ^]^ This form of agitation may be advantageous for the development of CM, as myofiber alignment and mechanical strength are essential characteristics.

A hollow fiber bioreactor has been used to promote the proliferation of skeletal muscle cells.^[^ ^163^, ^164^ ^]^ The classification of hollow fiber bioreactors as hydraulic bioreactors indicates that mixing is accomplished by liquid flow rather than mechanical mixing. This entails seeding the cells in a matrix of porous hollow fibers so that they adhere to the surface of the fibers, where the medium can also circulate. A hollow fiber system has the advantage of producing minimal shear stress and allowing for a greater variety of nutrients to be carried, making it excellent for highly metabolic cell types. Another bioreactor type is air lift, which achieves mixing using gas purging and may be useful for meat production.^[^ ^165^ ^]^ However, it lacks the record of accomplishment of other bioreactor designs that have been improved for several large‐scale bioprocesses.

Several cellular parameters must be evaluated when designing a bioreactor system to produce CM. Several kinds of meat cells, including myocytes, are anchorage dependent and must attach to a surface to proliferate and differentiate appropriately. Before differentiating into specialized cell types that require anchoring, it may be possible to grow the initial cell source in suspension. Alternately, growth methods employing nonadherent free‐floating spherical aggregates may be beneficial to avoid the potential requirement for a substrate during bioreactor development.^[^ ^165^ ^]^ This culture method would be more applicable to sources of PSCs that can be cultivated as free‐floating aggregates. Other adult stem cell sources, such as MSCs and muscle satellite cells, necessitate attachment substrates. There is also a risk of necrotic core development if the aggregates become too large and limit nutrition and oxygen passage. Considerations should also be given to the idea of co‐ccultivating muscle and fat cells to obtain CM. Today, however, it is still challenging to perfect the growth media that can support both cell types; it is likely that the two cell types will be cultivated separately soon. Perfusion bioreactors, which combine continuous medium input with perfusion flow, are a method for producing meat products of a certain size.^[^ ^166^, ^167^ ^]^ This is due to perfusion flow rate in these bioreactors that can be adjusted to the shape and size of the cultivated tissue. However, it should be noted that an increase in perfusion flow rate in proportion to the size and scale of the scaffold may lead to elevated shear stress and reduced pressure, potentially causing cellular harm. Certain bioreactor systems may be excellent for producing one form of CM, but they may not be suited for the development of other types and sizes of meat. As the field expands and seeks to meet a wide variety of CM products, bioreactor systems for large‐scale production will need to be continually optimized.

## 3 Insect Cells as a Source of Cells for Cultivated Meat

Cells sourced from a variety of species, principally bovine, porcine, and avian, have been targeted for the development of CM. Cells derived from less common species may be useful in overcoming current technological challenges that prevent the development and extension of cell cultures, such as the need for adherent cells and the high cost of media. Mammalian cells require a set of specific growth conditions and tight process control to maintain their functions: pH range of ≈ 6.8–7.8, temperature range of 30–39 °C, CO_2_, specific antibiotics, expensive GFs, animal‐derived serum, and adhesion for growth. Although some media formulations exclude serum, mammalian cells are difficult to adapt or thrive in serum‐free conditions.^[^ ^168^, ^169^ ^]^ Furthermore, most CM‐relevant cell types require adherent cultures, constraining growth by surface area. These limitations render large‐scale production of mammalian‐based cell culture systems difficult and inefficient.

In contrast, insect cells have properties that indicate suitability for large‐scale production in a more cost‐efficient manner. The use of insect cell culture for food applications has been summarized recently^[^ ^170^ ^]^ and will be briefly discussed here. Insect cells can tolerate a wide range of environmental variables, including pH (6.0–7.0) and temperature (20–32 °C), and are typically grown without CO_2_. The immortalization process can also occur spontaneously, as demonstrated by several insect cell lines in the Cellosaurus database and one cell line derived from *Manduca sexta* have been explored for food purposes.^[^ ^171^, ^172^ ^]^ Insect cells have the flexibility to grow either in suspension or attached to surfaces because they are not affected by contact inhibition. This means they can be cultivated in suspension bioreactors, where factors like surface‐to‐volume ratio and cellular biomass concentration can be finely tuned within a confined space.^[^ ^123^, ^124^ ^]^ A further difficulty with mammalian cell culture is the amount of culture medium required to support cells because of the high glucose consumption rates and toxic byproduct accumulation during cell expansion. In the context of cellular agriculture, this is a focal point of ongoing research as cost‐effectiveness is crucial for consumer adoption of CM products, and media is a large contributor to production costs. In contrast, insect cells produce fewer toxic byproducts like lactic acid, due to their metabolic processes, they exhibit lower sensitivity to toxic compounds (e.g., ammonia, a byproduct of catabolism), and they require less glucose for growth, thereby reducing the cost of materials and the volume of culture media required. These characteristics result in reduced material costs and lower volumes of culture media necessary, simplifying and making production scalability more economical. Rubio et al. (2019) investigated the differences between mammalian and insect cell cultures, focusing on cost, maintenance, and adaptability. These characteristics are crucial for advancements in TE, particularly in applications such as biofabrication, biobots, and CM. **Table** **1** provides a detailed comparison between these two types of cultures, highlighting their respective advantages and limitations.^[^ ^173^ ^]^

**Table 1:** Comparative analysis of mammalian and insect cell cultures for CM. The table outlines key differences between mammalian and insect cell cultures, focusing on growth conditions, cost‐effectiveness, and adaptability, while highlighting the advantages and limitations of both sources.

	Mammalian cell cultures	Insect cell cultures
Advantages	–Widely used in medical and biotechnological research –Better consumer acceptance	–More adaptable to serum‐free media –Growth in the absence of CO_2_ –Withstanding adverse environmental conditions –Growth near at room temperature (20–32 °C) –Less nutrients requirement –Less frequent replacement of medium –Lower costs –Easier transition between adherent and suspension cultures
Limitations	–More glucose consumption –More lactic acid production and need for replacement of acidified culture medium –Not easily adaptable in serum‐free media –Growth in adhesion, due to contact inhibition, therefore needing large spaces –More susceptibility to environmental conditions –Necessity of controlled levels of CO_2_ –Need to maintain a temperature of 37 °C –Higher costs –Complexity of scalability	–Further studies are needed –Complexity of scalability –Low consumer acceptance

Currently, finding a reliable and scalable source of insect muscle and fat cells is a significant obstacle to the production of insect tissues for food. To appreciate how insect cell cultures may be utilized to make CM, it is essential to understand the physiology of the cell types of interest. The mechanisms described are advantageous because they provide a method to explore in vitro the key biological properties of insect muscle and fat cells. Nonetheless, establishing stable lines of muscle and adipogenic progenitor cells capable of protracted proliferation and possible differentiation are essential for the manufacturing process and its scalability.^[^ ^174^ ^]^

### 3.1 Muscle Cells

Key differences between mammalian and insect muscle development include the origin and types of muscle cells, the molecular pathways that regulate them, and the function of muscle in the body. **Table** **2** offers an intricate comparative analysis concerning cell types, development, and molecular pathways. For an extensive review of primary myogenic insect cell culture attempts, readers are referred to Rubio et al. 2019.^[^ ^175^ ^]^ It has been suggested that CM production can help with environmental and animal welfare issues. While establishing bioproduction methods from mammals has been the focus of academic research on cell‐CM, it would be preferable to begin with relevant animal species like insects. More study is required to determine whether the pattern observed in mammalian cell types, in which cell multiplication decreases as animals age, is also reflected in the relationship between the age of insects and their proliferative ability.

**Table 2:** Comparison of muscle cells in mammals and insects. The table compares the characteristics of muscle cells in mammals and insects, highlighting differences in cell types, development, and molecular pathways and analyze the unique structural and functional distinctions in their muscle systems.

Types of Muscle		
Characteristics	Mammals	Insects
Classification	Divided into skeletal, cardiac, and smooth muscle.	Divided into skeletal and visceral; primarily striated muscle, only a small part lining the internal organs is smooth type.
Cell structure	Skeletal and cardiac muscles have striations, while smooth muscle does not. Skeletal muscles can be large and multinucleated.	Similar striated structure, with skeletal muscles attached to the exoskeleton; visceral muscle is less numerous.
Contractile units	Actin and myosin in sarcomeres.	Like mammals, actin and myosin are organized in sarcomeres.
Number of nuclei	From mononucleate to multinucleate depending on the type of muscle.	From binucleate to multinucleate, often fewer nuclei are compared to mammals.
Muscle Development		
Development process	Mammals	Insects
Differentiation	Differentiation from satellite stem cells to myofibers, using GFs such as FGF.	Formation from myoblasts (skeletal muscle precursors), which divide into founder cells and fusion‐competent myoblasts.
Regeneration	Presence of satellite cells for the regeneration of skeletal muscles.	Limited regeneration; some insects regenerate muscles during metamorphosis.
Interaction with other tissues	Interactions with the nervous system for innervation and regulation.	Essential interactions with neurons for complete development.
Molecular Pathways		
Molecular regulation	Mammals	Insects
Regulatory factors	MyoD, Myf5, myogenin, MRF4, CTNNB1, and GSK3B are crucial for muscle development. FGF, IGFs, myostatin, TGF regulate proliferation and differentiation.	Twist, a class of bHLH factors, regulates the distinction of myoblasts. Notch and Ras/MAPK pathways for the selection of muscle precursors.
Hormonal regulation	Hormones such as IGF, GH, and sex hormones are important for muscle growth.	Ecdysone, induced by PTTH, and juvenile hormones regulate growth and metamorphosis.

The demands that have given rise to the establishment of this new frontier may be addressed through understanding of the features of muscle and adipogenic growth. Additionally, controlling the creation of muscle or fat in vitro would require knowledge of the signals that govern these processes in vivo, and mammalian indications are not suitable for insect cells.

#### 3.1.1 Muscle Types

Several cell types that are all specialized for contraction are referred to as “muscle.” Despite their other differences, all muscles share the same metabolic processes that drive contractions. This process involves the interaction of actin and myosin proteins within the muscle fibers, with ATP serving as the primary energy source.^[^ ^176^ ^]^

In vertebrates, the broadest classification of muscle is based on the presence or absence of regular cross striations. In mammalian systems, there are three main types of muscle: skeletal, cardiac, and smooth, which has no striations. Skeletal muscle cells can be enormous (up to half a meter long with a diameter of 100 μm in adult humans) and are often called muscle fibers because of their elongated shape. Each cell constitutes a syncytium containing many nuclei immersed in the same cytoplasm. Other types of muscle cells are more conventional in that they possess a single nucleus.^[^ ^176^ ^]^

The walls of numerous organs and tubes in the body are lined with layers of smooth muscle cells, and smooth muscle does not contract voluntarily. The smooth muscle cells shorten when forced to contract, driving the organ's luminal contents, or the cell shortening changes the diameter of a tube to control the flow of its contents. Smooth muscle cells lack the striated banding pattern present in cardiac and skeletal muscle and are neurally innervated by the autonomic nervous system. In addition, hormones, autocrine/paracrine substances, and other regional chemical signals regulate the contractile state of smooth muscle.^[^ ^177^ ^]^ The thick central layer of the heart is made up of cardiac muscle (also known as myocardium). The individual cells that make up the heart muscle are known as cardiomyocytes. Cardiomyocyte main function is to contract in order to create the pressure required to pump blood through the circulatory system.^[^ ^178^ ^]^ Each cardiac muscle cell, or cardiomyocyte, is a tubular structure made up of chains of myofibrils, which are rod‐like components inside the cell. Sarcomeres, the primary contractile units of muscle cells, are repeated in sections to form the myofibrils. Long proteins that form myofilaments, or thick and thin filaments, make up sarcomeres. Actin is a protein found in thin myofilaments, whereas myosin is a protein found in thick myofilaments. As the muscle contracts and relaxes, the myofilaments move past one another. When seen under a microscope, the arrangement of thin and thick myofilaments overlapping within the cell' sarcomere gives the illusion of being striated, similar to that of skeletal muscle.^[^ ^179^ ^]^ All insect muscles follow a similar structure, with elongated cells holding the contractile components and frequently inserting into the integument at each end. But various muscles have diverse internal arrangements of the muscle cells, and wing muscles frequently have distinctive shapes.^[^ ^180^, ^181^ ^]^

Insect muscles are almost all striated and are divided into two groups: skeletal muscles and visceral muscles, only a small part of which are of the smooth type, lining the walls of internal organs. Skeletal muscles can be attached to the exoskeleton internal surface area in significantly greater numbers than can fit on the skeletal framework of vertebrates. Elongated contractile fibers that are parallel to or converge at the point of insertion form the skeletal muscles. A consistent network of longitudinal and circular fibers can develop in visceral muscles.^[^ ^182^ ^]^ Somatic muscles (or of the body wall) of insects like *Drosophila melanogaster* do not have many muscular fibers like those found in mammals. Additionally, the 30 segmentally repeated muscle fibers that make up somatic muscles are arranged in a clear pattern. The body wall muscles of many insects have from 4 to 24 nuclei, compared to up to 1000 nuclei in mammalian muscles. The visceral muscles that surround the gut, in addition to the somatic muscles, are syncytial. Circular binucleate muscles with partial fusion and multinucleate longitudinal muscles make up the larval midgut muscles. In the flight muscles, quiescent satellite cells have been identified that, as in mammals, can be activated by injury.^[^ ^183^ ^]^

#### 3.1.2 Muscle Development

As already mentioned, prototypes of CM products have focused on the differentiation of stem cells, such as muscle satellite cells, to produce skeletal muscle, which is the main component of traditional meat. Consequently, our focus will be on the development of skeletal muscle. Myogenesis, the process of muscle formation, is substantially conserved in both invertebrate and vertebrate species. The embryogenic myogenesis of *D. melanogaster* is a well‐recognized model for studying the genes and mechanisms that drive muscle development.^[^ ^76^ ^]^

Myoblasts, precursors of skeletal muscle cells, fuse to form multinucleated cells after a proliferation period. Like somatic musculature, the muscles of the visceral mesoderm (circular and longitudinal muscles) of many insects are composed of founder cells and fusion‐competent myoblasts. The founder cells responsible for the development of the circular and longitudinal muscles originate from^[^ ^176^ ^]^ distinct regions within the mesoderm. The founder cells of the visceral circular muscles (cFC) and the myoblasts competent for fusion (cFCM) originate from the mesoderm of the visceral trunk, abbreviated as TVM.

Founder cells and fusion‐competent myoblasts are the two groups of myoblasts that fuse in a variety of insects. Founder cells express a particular combination of identity transcription factors that facilitate the identification of muscle fibers and define their orientation, shape, size, and attachment site. Fusion‐competent myoblasts, on the other hand, have a generic identity. They express the transcription factor Lame Duck (Lmd), but it is not yet clear how reprogramming occurs.^[^ ^183^ ^]^

During fusion, they undergo a profound change in phenotype that depends on the coordinated activation of a set of specific genes (see Section  “Molecular Pathway”). The regulatory protein is myoD1, normally expressed only in myoblasts and muscle cells. The experimental induction of myoD1 is also capable of transforming a fibroblast into a myoblast. The skeletal muscle cell, once formed, is generally preserved throughout the animal's life.

Some myoblasts persist in the adult muscle and appear as small, flattened, quiescent cells in close contact with mature cells within their basal lamina envelope. These satellite cells are activated to proliferate when the tissue is damaged or, for example, by artificially treating them with FGF. Myoblasts maintained in culture for up to 2 years retain the ability to differentiate and fuse to form muscle cells in response to appropriate changes in culture conditions. FGF is essential in keeping myoblasts in a proliferative state and preventing them from differentiating.^[^ ^176^ ^]^ Since there is evidence that insect myoblasts require direct interaction with neurons to fully develop, the absence of support cell types in the initial cultures may be the source of restricted differentiation in these isolated insect muscle cells that have been immortalized. For CM large‐scale production, the ability to control the proliferation and differentiation of cultured muscle cells is critical.

#### 3.1.3 Molecular Pathway

In mammalian systems, myoblasts are actively growing MPCs that are produced once a quiescent satellite cell is activated. Its proliferation is fueled by the myogenic regulatory factors (MRFs) MyoD and Myf5. Proliferation is aided by FGF, inhibited by myostatin and transformed GF (TGF). IGFs, or insulin‐like GFs, promote both proliferation and differentiation. Myoblast fusion into primary myofibers is fueled by IGFs and the MRF myogenin. IGFs and MRF4 encourage further fusion and differentiation, resulting in secondary fibers that eventually mature into myotubes with associated quiescent satellite cells. A second pair of MRFs, MRF4 and Myogenin, are increased during differentiation, promoting differentiation and fusion as well as assisting in maintaining the mature muscle structure.^[^ ^67^ ^]^ It has been demonstrated that CTNNB1 (‐catenin) and GSK3B (glycogen synthase kinase‐3) control the direction of skeletal myogenesis in animals like pigs from the earliest stages of embryonic development through terminal differentiation.^[^ ^61^ ^]^ The two types of myoblasts found in insects originate from mesodermal regions that exhibit elevated levels of the bHLH transcription factor Twist. The high Twist domain exhibits a distinct mechanism whereby a muscle progenitor cell is selectively identified through a crosstalk between the Notch and Ras/MAPK signaling pathway. The progenitor myoblasts undergo asymmetric division, resulting in the emergence of either two founder cells or a founder cell and an adult precursor cell (AMP). The latter, while in a state of quiescence and undifferentiation during embryonic development, undergoes reactivation in the second larval instar, ultimately leading to the generation of adult fly muscles. The myogenic cluster residual cells that exhibit Notch expression undergo differentiation into myoblasts that are capable of fusion.^[^ ^67^ ^]^

#### 3.1.4 Hormonal Regulation

In *D. melanogaster*, as in many other holometabolous insects, muscle formation occurs twice: during embryogenesis and metamorphosis. These insects show critical morphological differences between larval and adult stages. As a result, the muscles that develop during the embryonic stage of an organism are eliminated during the pupal phase of metamorphosis and substituted with adult muscles.^[^ ^183^ ^]^

Without the insertion of additional myoblasts or nuclear division within the muscle syncytium, muscles in the larval stage enter a degree of hypertrophy. The arrangement of adult muscles differs significantly from that of larval muscles. The embryonic mesoderm engages the precursors of the adult muscles and delays their differentiation. The myoblasts for the head and thorax muscles are retained in the imaginal discs until pupal development, when the muscles develop. The imaginal disc is one of the components of a holometabolous insect larva that will change during pupal metamorphosis into a section of the adult insect's exterior. There are disc pairs that may be used to create various structures, including wings, legs, antennae, and other parts.^[^ ^184^ ^]^ Most larval muscles are histolyzed to create adult muscles during metamorphosis, while adult muscles are de novo formed through the migration and fusion of adult MPCs.^[^ ^185^ ^]^

Insect muscle cells undergo profound changes during the animal's lifecycle. In this dynamism during growth, molting, pupation, and metamorphosis, certain hormones play an important role. In several species, the molt is stimulated by a hormone called ecdysone (also known as molting hormone). This hormone is secreted by two prothoracic glands, situated in the insect's thorax, and it is responsible for the growth and differentiation of adult structures. The production of ecdysone is in turn stimulated by a brain hormone, namely the prothoracicotropic hormone (PTTH).^[^ ^186^ ^]^

In holometabolous insects, complex signals control both the timing and developmental stage as the animal undergoes metamorphosis. The endocrine function that controls these stages has been extensively studied in several moths, including the silk moth (*Bombyx mori*) and the tobacco hornworm (*Manduca sexta*). Whether a molt leads to larva, pupa, or an adult depends on the presence or absence of juvenile hormone. The juvenile hormone is present in the earlier larval stages, and the larval molt leads to a bigger larva. In the last larval instar, the level of juvenile hormone falls sharply, and pupa is formed. The final molt, when the pupa develops into an adult, depends on the absence of juvenile hormone.^[^ ^186^ ^]^ Despite a less comprehensive understanding of hormonal pathways in insect muscle development compared to humans and other animals, significant advancements have been achieved in differentiation and proliferation. Low doses of 20‐hydroxyecdysone stimulate myoblast proliferation in *M. sexta*, but concentrations beyond the critical threshold inhibit myoblast growth. Methoprene, an analogue of juvenile hormone, inhibits the capacity of high doses of ecdysteroid (Ecd) to induce proliferative arrest and differentiation.^[^ ^187^ ^]^ The hormonal regulators of the differentiation process can be employed to regulate cell growth throughout production.^[^ ^188^ ^]^

In mammals, primary muscle‐resident progenitor cells isolated from skeletal muscle differentiate into smooth and skeletal muscle, whereas satellite cells only differentiate into skeletal muscle. Differentiation in culture is based on using biological or chemical substances in cell culture media.^[^ ^189^ ^]^ Proliferation, differentiation, and fusion processes are associated with the activity of several known hormones, GFs, and transcription factors. The pathways in which they are involved play a crucial role in controlling muscle growth, energy metabolism, and repair of damaged muscle tissue. Some of the most important hormones involved in muscle development include testosterone, insulin, growth hormone (GH), IGF‐1, cortisol, and other thyroid hormones. The molecular control mechanisms that direct skeletal muscle development have significant implications for medicine, agriculture, and food technology.^[^ ^61^ ^]^ In addition to their biological functions, the genes involved serve as important markers for monitoring and optimization in CM applications.^[^ ^67^ ^]^

### 3.2 Fat Cells

In cellular agriculture, fat is essential for flavor and nutrition. To provide healthy and tasty food, both muscle and adipose tissue are needed. Many differences can be detected between the adipose tissue of mammals and that of insects, which vary in terms of their cellular composition, regulation, function, and anatomical location. **Table** **3** summarizes numerous distinctions observed.

**Table 3:** Comparison of mammalian and insect fat cells. The table delineates a comparative analysis of fat cells in mammals and insects, elucidating their unique characteristics, functional roles, and potential applications in cellular agriculture for food production.

Composition and Development		
Aspect	Mammalian fat cells	Insect fat cells
Origin	Derived from MSCs in the mesoderm during embryonic development.	Located in the hemocoel, with various origins specific to insect physiology.
Types and functions	White adipocytes for energy storage, primarily in subcutaneous and abdominal areas; brown adipocytes are specialized for thermogenesis.	Trophocytes for nutrient storage and metabolism; enocytes for carbohydrate synthesis; mycetocytes, symbiotic prokaryotic microorganisms for nutrient synthesis; chromatocytes for lipid storage to support metamorphosis; urocytes for storing urate granules.
Appearance and Features	WAT has a large, circular shape, adipocytes unilocular; BAT, adipocytes multilocular, rich in mitochondria and with a high concentration of blood vessels, which contribute to the brown hue.	Cells are versatile in function, not specifically color coded but distinguished by their specific roles and content (such as lipid, protein, and carbohydrate).
Function and Regulation		
Aspect	Mammalian fat cells	Insect fat cells
Regulatory mechanisms	Governed by hormones such as insulin (energy storage), leptin (appetite control), and adiponectin (glucose and lipid metabolism). Bioactive lipids from BAT promote glucose uptake and thermogenesis.	Regulated by a range of hormones like AKH (energy mobilization), Ecd (metamorphosis), juvenile hormone (growth and development), which impact a wide array of physiological processes from growth to systemic immunity.
Research and Applications		
Aspect	Mammalian Fat Cells	Insect Fat Cells
Cultivation for food	Studies focus on deriving adipocytes from pluripotent and MSCs and DFATs for CM applications. Commonly used cell lines include 3T3‐L1 and other murine lines for research and food production. Continued exploration into efficient and scalable methods to cultivate mammalian adipose tissue in vitro, aiming at texture and taste that mimic natural meat.	Emerging research into cultivating insect fat cells, notably for their roles in nutrient storage and release which can enhance muscle cell co‐culture systems. Specific culture conditions are being developed to optimize lipid accumulation and usage in sustainable food production systems.

#### 3.2.1 Mammalian Fat Tissue Composition

In mammals, adipose tissue is a connective tissue that primarily serves as a lipid storage of food and energy, as well as providing a significant amount of heat, water, and thermal insulation. Adipose tissue derives from MSCs that form during embryonic development from the mesoderm.^[^ ^190^ ^]^ Adipose tissue also plays a role in the body's metabolism through the production of hormones, cytokines, proteins, and peptides. In mammals, adipose tissue is composed of white adipocytes (the primary site for energy storage) and brown adipocytes (specialized in thermogenesis). The predominant lipid‐containing tissue in mammals is white adipose tissue (WAT), also known as unilocular adipose tissue. The location of WAT tissue is predominantly in the subcutaneous and abdominal region, and prominent deposits are also observed in skin and bone.^[^ ^191^ ^]^ WAT is predominantly found in the mesentery and intraperitoneal, with a lesser presence in the bone marrow and surrounding the visceral organs. The subcutaneous adipose tissue, apart from serving as a reservoir of energy, functions as a thermal insulator against low temperatures.

WAT adipocytes are circular in shape and possess a significant size, measuring over 100 μm in diameter. They are characterized by a substantial lipid droplet that occupies most of their internal space. Adipocytes that are unilocular in nature are segregated by the slender strata of lax connective tissue, which are replete with reticular fibers that are secreted by the adipocytes themselves. Furthermore, it should be noted that every adipocyte is enveloped by a slender coating of ECM, which is situated near the plasma membrane. The outer lamina, which bears resemblance to the basal lamina of the epithelium, and is distinct from the adjacent connective tissue, is referred to as the sheath. In addition to mature white adipocytes and septa, various other cell types, including mast cells, macrophages, leukocytes, dispersed fibroblasts, and undifferentiated adipocytes, can also be observed. The dermal deposit and subcutaneous deposit are distinct entities that are physically segregated. Brown adipose tissue (BAT), also known as multilocular adipose tissue, is due to the presence of adipocytes that possess numerous small lipid droplets within their cytoplasm. It is prevalent in hibernating species, developing fetuses, and mammals during the perinatal period. BAT emerges prior to WAT during the developmental process. Brown adipocytes are smaller than white adipocytes and possess a rounded nucleus that is situated in the central regions of the cytoplasm. The abundant presence of cytochrome oxidase within the mitochondria of adipocytes is responsible for the brown hue exhibited by fresh BAT. The high concentration of blood vessels within the tissue is also a contributing factor to the brown hue. BAT adipocytes are distinguished from WAT adipocytes by the presence of the UCP1 protein, which serves to disengage the chain of electron transporters from ATP synthesis. This results in the utilization of the proton gradient energy for the purpose of heat generation.^[^ ^191^ ^]^

#### 3.2.2 Insect Fat Body Composition

Numerous insect species have high quantities of essential fatty acids such as omega‐3 and omega‐6.^[^ ^192^ ^]^ In addition to lipids, the fat body tissue of insects contains proteins and carbohydrates.^[^ ^193^ ^]^ The insect fat body is essential to metabolic processes. It is situated in the hemocoel, where its constituent cells are near the haemolymph, allowing the exchange of metabolites. The fat layer just below the body wall is typically peripheral or parietal, while the layer that surrounds the feeding channel is frequently perivisceral. Although parts of the fat body also extend into the chest and head, the majority is in the abdomen. Fat body shape can vary widely between orders and species. In hemimetabolous insects, the larval fat body remains mostly unchanged in the adult form. In holometabolous insects, the fat body goes through a remarkable metamorphosis in which the tissue separates into individual cells. The adult adipose cells in the majority of holometabolous insects come from the larval adipose cells, but the adult fat cells in the Hymenoptera and higher Dipterans are created entirely de novo.^[^ ^194^ ^]^ The storage of body fat plays a fundamental role in the lifecycle of holometabolous insects. Throughout the larval feeding stages, energy stores accumulate to facilitate the metamorphic process and create reserves for the emerging adult organism.^[^ ^195^ ^]^ Furthermore, the quantity of nutrients accumulated in the larvae has significant implications for their adult life, as diminished larval fat body size leads to decreased reproductive capacity.^[^ ^196^ ^]^ Mature arthropods that exhibit a nonfeeding lifestyle depend on these endogenous reserves to sustain their vital functions and reproductive activities. The process of egg development necessitates a significant transfer of resources from the adipose tissue to the ovaries. The significance of fat body reserves transferred from larval stages for oogenesis is evident in autogenic mosquitoes. In these mosquitoes, the activation of the target of the rapamycin signaling pathway and the subsequent maturation of eggs following a blood meal depend on the accumulation of sufficient nutritional reserves during larval development.^[^ ^197^ ^]^

In insects, the fat body is composed of five distinct cellular subtypes, exhibiting heterogeneity in their composition, dimensions, and functions and physiological roles across various developmental phases. Trophocytes are the most abundant cells. These cells are primarily responsible for the retention, excretion, and elimination of organic compounds. The cells exhibit variability in both size and quantity. Alterations in size are attributed to the accumulation of numbers and the expansion of the vacuoles. Four distinct types of vacuoles can be identified: digestive vacuoles, which facilitate metabolism and nutrient release during energetic or diapause expenditure; and storage vacuoles, which are responsible for the storage of reserve substances. Condensation vacuoles are associated with the Golgi apparatus and lysosomes and typically harbor proteins and surface vacuoles that arise from the fusion of vesicles (as observed in cellular specimens). The quantity of trophocytes is subject to variation, in addition to variations in their structural composition. Male insects exhibit a lower count of trophocytes in comparison to their female counterparts. Additional cells are also observed during the process of molting.^[^ ^198^ ^]^

Enocytes are a type of cell that exhibits a circular or oval shape and are commonly found in association with the epidermal layer of the cuticle. These cells may also be present alongside the predominantly parietal adipose body. Enocytes possess a nucleus that is situated centrally, along with mitochondria, smooth endoplasmic reticulum, and vacuoles that contain lipid, protein, and glycogen droplets and granules. These cells are capable of synthesizing carbohydrates that are transported between the hemolymph and body fat.^[^ ^199^ ^]^

Mycetocytes are cellular entities primarily composed of symbiotic prokaryotic microorganisms. They co‐exist in a perpetual state of symbiosis with insects in a certain quantity. Mycetocytes are observed at the level of cytoplasmic fat and glycogen granules. Mycetocytes are present in nutrient‐deficient and imbalanced environments and are responsible for the biosynthesis of certain essential nutrients, including amino acids and B‐group vitamins.^[^ ^200^ ^]^

Chromatocytes are thin cells that show a central nucleus and a clear cuticle. These cells are situated in the thinnest layers of the fat body and accumulate lipids to support metamorphosis. Chromatocytes are present in select species of aquatic insects. Finally, urocytes exhibit distinctive features such as a diminished endoplasmic reticulum and a vacuole containing urate granules. Urate is derived from either the metabolic breakdown of nucleic acids or the degradation of proteins. The primary function of these cells is to accumulate and retain urate granules.^[^ ^201^ ^]^ The fat body was one of the earliest forms of insect tissue to be cultivated in vitro for the study of protein production.^[^ ^202^ ^]^ Important proteins, such as vitellogenin, the precursor protein of the egg yolk, and growth hormones that bind to proteins, are generated by fat body cells.^[^ ^203^, ^204^, ^205^ ^]^ Fat–tissue‐specific cells grow slowly at first but can be formed into continuous lines.^[^ ^206^ ^]^ Other insect cells can be grown in vitro using fat body cells: the accumulation and release of nutrients by fat cells can extend the survival and contraction of muscle cells in vitro for months without altering the medium.^[^ ^207^ ^]^ Similarly, the development of embryos in vitro can be improved through the fat cells.

#### 3.2.3 Fat Tissue Function

Adipose tissue in mammals serves various functions, including acting as a crucial mediator of metabolic control and communication, regulating thermoregulation, providing protection against cold and trauma, and controlling reproduction and satiety.^[^ ^190^ ^]^

The insect's fat body, instead, is a versatile organ that performs a variety of physiological functions, including metabolic regulation, signal integration, regulation of molting and metamorphosis, and synthesis of hormones that modulate systemic function and immune protein synthesis. The substrates and products of numerous pathways in fat cells include lipids, carbohydrates, and proteins, which can serve as sources of energy production, reserves, and mobilization during various stages of life such as diapause, metamorphosis, and flight. The adipose tissue also acts as the primary site for the integration of innate and adaptive humoral immune responses, as it is primarily responsible for the synthesis of antimicrobial peptides.

Throughout the insect's life cycle, the adipose tissue undergoes a sequence of modifications, including development, expansion, and restructuring in the embryonic, larval, and pupal stages, while also governing reproductive processes in the adult stage. These alterations and regulatory mechanisms are regulated by hormonal and nutritional signals.^[^ ^201^ ^]^

#### 3.2.4 Fat Tissue Regulation

In mammals, the regulation of metabolism in adipose tissue is primarily governed by insulin, which serves as the catalyst for the absorption and storage of energy.^[^ ^208^ ^]^ Leptin is an additional hormone that doesn't cause feelings of satiety. However, decreased levels of leptin serve as an indicator of reduced energy reserves, leading to an increase in appetite and the desire to consume food.^[^ ^209^ ^]^ The regulation of glucose and lipid metabolism within mammalian fat tissue is attributed to Adiponectin, which also facilitates a metabolic profile that exhibits antiatherogenic, anti‐inflammatory, and insulin sensitizing properties.^[^ ^210^ ^]^ The identification of molecules that have effects has contributed to the progress in comprehending adipose tissue as an endocrine organ. Bioactive lipids, including 12,13‐dihydroxy9Z‐octadecenoic acid (12,13‐diHOME) and 12‐Hydroxyeicosapentaenoic acid (12‐HEPE), are secreted by BAT and promote the uptake of glucose and fatty acids in both BAT and skeletal muscles, thereby facilitating sustained thermogenesis.^[^ ^211^ ^]^

The metabolism of the fat body in insects is governed by a multitude of compounds, enzymes, and substances, primarily through the influence of hormones that modulate the activity of metabolic processes within the adipose tissue. Hormonal activity plays a crucial role in the process of insect metamorphosis, including the development and timing of molting. The hormones that commonly regulate various processes include adiponectin AKH, Ecd), juvenile hormone (JH), neuropeptide activating the diapause‐pheromone hormone biosynthesis (DH‐PBAN), corazonin (crz), leucochine (Lk), CCHa2, allanostatin‐A (Ast ‐A), tachykinin (Tk), limostatin (Lst), cytokines, short neuropeptide F (sNPF), and neuropeptide F (NPF). The neurosecretory cells in the heart bodies create the peptide known as AKH.^[^ ^212^ ^]^ Additionally, AKH is expressed in the middle intestine, muscle, body fat, and ovaries. It is comparable to glucagon and has 8–10 amino acids. Numerous insects have several AKHs, and migrating locusts have three variations with distinct bioactivities.^[^ ^213^ ^]^ The hormone is originally present as a prohormone that splits off as AKH from the peptide associated with the adipokinetic hormone precursor (APRP) when it is activated. Due to the management of energy stores and their mobilization in insects’ bodies during mutation and metamorphosis, the activity of AKH is present at the most crucial developmental phases.^[^ ^214^ ^]^ It largely affects how enzymes such as glycogen phosphorylase, which converts glucose into sugars, and triglyceride lipase, which is involved in lipid metabolism. A common reaction to abrupt changes in lipid levels is the formation of AKH. The transduction signal AKHR (i.e., AKHR) activates the hormone. After that, phospholipase C, which converts membrane lipids into inositol 1,4,5‐trisphosphate and diacylglycerol, is activated by AKHR. AKHR influences inositol triphosphate (IP_3_) concurrently, which elevates calcium ions in the endoplasmic reticulum and transfers them to the cytoplasm. In addition, the activation of the hormone impacts the commencement of the activity of adenylate cyclase and hence the generation of cAMP. As a result, AKH activation controls the amount of TG in the fat body.^[^ ^215^ ^]^ The hormone raises heart rate, motility, and neural signaling; it improves muscular tone; and it protects against oxidative stress. It also affects CREB, calcium homeostasis, and the expression of genes related to fat degradation.^[^ ^215^, ^216^ ^]^

JH regulates various processes that affect the larva's growth and appearance and promotes the production of vitellogenin, a crucial precursor to the bull protein that is delivered into the ovocytes. It has been demonstrated that JH regulates protein granule existence and that its absence signals metamorphosis by causing the cytolysis of the larval body fat and the synthesis of a new one. The counterpart JH‐1 also results in the vacuolization of aged trophocytes. This hormone low content induces the formation of vitellogenin in the fat body.^[^ ^217^, ^218^ ^]^

Ecd functions in opposition to and concurrently with JH. By promoting tissue dissociation (the metamorphosing stage), tissue remodeling, and the emergence of autophagic structures. Ecdysone controls the timing of metamorphosis. JH prevents premature aging and transformation. Serious deformities, issues with mutation, and a lack of transformation are brought on by a deficiency of any of these hormones.^[^ ^218^ ^]^

The intricate nature of hormonal regulation in insects suggests that hormonal signals may have an impact on the storage of lipids in fat cells, and therefore, it is recommended that in vitro fat body culture includes supplementation with such signals. While the application of these systems in mammals is well understood and can be readily applied in the CM industry, the understanding of these systems in insects is comparatively limited, necessitating further research.

Currently, most of the experimental research on cellular agriculture has prioritized the production of muscle cells, given their prominence in the biomass of meat products. Nevertheless, it is widely recognized that fat content plays a crucial role in determining the taste, consistency, nutritional value, and consumer acceptability of cellular meat. The optimal source to produce cultivated fat remains uncertain. However, in mammals, several cell types exhibit the ability to undergo adipogenic differentiation in vitro.^[^ ^219^ ^]^ PSCs represent a potential source for the generation of mature adipocytes through successive differentiation. PSCs have been derived exclusively from ESCs originating from blastocysts of various animal species, including pigs and cows.^[^ ^220^, ^221^ ^]^ Adipocytes may be derived from MSCs, which are typically extracted from adipose tissue and bone marrow. Several studies have demonstrated the feasibility of extracting adipocytes from the larval phases of various insects. This involves dissecting the fat body from the abdomen and subsequently mincing it in a suitable culture medium.^[^ ^170^ ^]^ However, further research is necessary into methods for large‐scale production and control of lipid accumulation in insect fat body cells.

Several studies have demonstrated the possibility of co‐culturing muscle cells and insect fat cells. Particularly, the functions performed by fat cells, such as nutrient storage and release, have been found to enhance the survival and contraction of muscle cells. Additionally, it has been observed that both cell types can be cultivated using the same culture medium, which is a challenging task for mammalian cells due to the distinct media formulations required by the two cell types.^[^ ^207^, ^222^ ^]^ To create edible and nourishing food items, it will be crucial to cultivate both fat cells and muscle cells.^[^ ^170^ ^]^

## 4 Economic, Environmental, and Nutrition Sustainability of CM

Using TE techniques, in vitro CM allows meat production without the use of animals. In vitro CM may be more advantageous than traditional meat production in terms of costs, health, animal welfare, and the environment impact.^[^ ^223^, ^224^ ^]^ On August 5, 2013, in London, an in vitro beef burger was first publicized and tasted. Since then, the media has presented cell‐based meat as a novel approach to generate meat with enormous opportunity.^[^ ^221^ ^]^ Insects, as a potential alternative source of protein, also play a role in discussions about sustainable nutrition. It was possible to make a comparison between the nutritional profile of edible insects and mammalian meat. The Orkusz study^[^ ^225^ ^]^ compares the nutritional value of insects with that of meat from slaughtered animals, highlighting that both are rich in proteins, essential amino acids, unsaturated fatty acids, vitamins, and minerals. Although it is not possible to definitively state that insects are nutritionally superior to meat, some insect species show higher energy and specific nutrient contents, such as proteins and polyunsaturated fatty acids, compared to meat. Insects also have higher levels of certain minerals and vitamins and are a source of vitamin C and fiber, which are not present in meat. These nutritional characteristics make insects a potentially valuable resource for CM production to enrich diets and improve health, contributing to the fight against global malnutrition. A significant benefit of CM production is improved control over flavor, fatty acid composition, fat content, and the ratio of saturated to polyunsaturated fatty acids, by modifying the culture medium composition or coculturing with other cell types. In vitro CM does not require killing animals, and animal suffering as well as the number of animals used in meat production are projected to decrease because of in vitro CM; in theory, the supply of meat for the entire planet could be produced by a small farm that provides occasional biopsies.^[^ ^226^ ^]^ Ten stem cells could produce 50 000 metric tons of beef, if they divide and differentiate continuously for 2 months. Although in practice much optimization is needed to get to this level of efficiency,^[^ ^227^ ^]^ furthermore, strict quality control regulations, which are impossible to implement in contemporary animal husbandry, slaughterhouses, or meat packing plants, could significantly reduce the likelihood of meat contamination and the incidence of zoonotic diseases in large‐scale CM facilities. Additionally, traditional meat exposure to hazards like pesticides, arsenic, dioxins, and hormones should be significantly reduced.^[^ ^182^ ^]^ The in vitro cultivation of meat allows faster production of the final product compared to livestock, focusing on key meat components such as muscles while avoiding the production of unnecessary tissues like bones, respiratory organs, digestive organs, skin, and nervous system. In traditional meat production systems, a significant portion of the food consumed by animals does not effectively transform into meat due to metabolism and the formation of inedible parts like bones and brain tissue. In contrast, lab‐grown meat is time and energy efficient, taking only a few weeks instead of months (for chickens) or years (for pigs and cows) to be ready. Moreover, producing meat in vitro from insect cells has many environmental advantages linked to the differences from mammalian cells.^[^ ^170^ ^]^ In vitro CM will also significantly minimize land needs. The carbon footprint of meat products should also be reduced through in vitro manufacturing, which can also reduce greenhouse gas emissions from raising livestock for meat by up to 90% and land and water resources for raising meat by up to 80%, although further research and development is necessary to support these estimates during large‐scale CM production.^[^ ^228^ ^]^ The substantial decrease in land use projected creates opportunities for other uses of the land, such as reforestation, which may help in the recovery of many endangered species. The scientific, environmental, and animal rights sectors are also supportive of in vitro meat production because it is a more environmentally friendly method of manufacturing meat with fewer negative impacts on human health. By cultivating cells from rare or endangered animals held in captivity, or even cells retrieved from samples of extinct creatures, it would be possible to create new types of meat and meat‐based products for future markets, effectively enabling their consumption without impacting current populations. In many instances, such as those involving space missions, polar stations, troop encampments in remote theatres of war, and bunkers intended for long‐term personnel survival after nuclear or biological attacks, it may be more effective to produce food on‐site. In vitro meat production is a potential solution in these circumstances. In particular, the European Space Agency (ESA) is looking for ideas into how cellular agriculture could be used to grow food on long‐term space missions. This will reduce the quantity of perishable food that must be transported, give an alternate source of nourishment, and provide fresh food. Such a novel food production system for space should be included in a closed‐loop arrangement so that resources, especially the growth medium, may be recycled or regenerated, thereby minimizing dependence on supplies from Earth.^[^ ^229^ ^]^

In vitro meat production has many supporters, but also raises concerns. The unnatural nature of in vitro meat is an issue with adoption by the public and appears to be a factor in opposition to new food technology, at least in Europe.^[^ ^230^ ^]^ Novel foods are supposed to be essential to the shift to sustainable food systems. Nevertheless, whether and how much they are adopted into the diets of the public will determine their success. Frameworks for the production and distribution of CM are starting to be developed by governments and regulatory agencies. Having clear guidelines can help to increase consumer confidence and trust. According to research in the literature, the main obstacles to the adoption of CM include contextual issues like price,^[^ ^231^ ^]^ emotions like fear^[^ ^232^ ^]^ and disgust, and cognitive problems like lack of familiarity.^[^ ^233^ ^]^ In response, several tactics have been put out to boost consumer acceptance of CM. These tactics include educating customers about the production method and the advantages of CM, as well as facilitating production scale up^[^ ^234^ ^]^ point to present the product at a lower price. This is going to be a major motivator for consumer adoption.^[^ ^235^, ^236^ ^]^ CM advocacy will be greatly aided by influencer collaborations and educational efforts, and it should become a common choice for people looking for ethical, sustainable, and healthy food options as the sector develops.

## 5 Conclusion

CM has the potential to provide consumers with the nutrition they need while significantly reducing the animal suffering, environmental, and human health issues associated with conventional meat farming.

Due to the differences between insect and mammalian cells, in vitro CM from insects has considerable advantages for the environment and for large‐scale production with a more cost‐efficient approach. These include 1) the adaptability of insect cells to both adherent and suspension growth; 2) cost reduction of culture media. Mammalian cells require larger amounts of culture medium and components due to the high rates of glucose consumption and accumulation of toxic byproducts during cell expansion. Insect cells consume less glucose during growth, accumulate less lactic acid due to slower cell metabolism, and are less sensitive to toxic compounds. 3) tolerance of a wide range of environmental variations, including pH (6.0–7.0) and temperature (20–32 °C); and 4) they typically grow in the absence of CO_2_, and the immortalization process can occur spontaneously.

## Conflict of Interest

The authors declare no conflict of interest.

## Authors Contributions

F.G. and S.O. contributed equally to this work. Conceptualization was taken care of by P.F. Writing and the original draft preparation were taken care of by P.F., F.G., C.S., and S.O. Writing, review, and editing were taken care of by P.F., F.G., C.S., S.O., V.P., D.I., S.L., R.S., A.L.G., and D.K. Supervision was taken care of by PF.
